# Causal association of smoking and laryngeal cancer: A Mendelian randomization study

**DOI:** 10.18332/tid/209744

**Published:** 2025-11-21

**Authors:** Fengbo Yang, Xing Chen, Ruoying Wei, Ping Lv, Mohammed Abdelfatah Alhoot

**Affiliations:** 1 Department of Otolaryngology, Affiliated Hospital of North Sichuan Medical College, Nanchong, China; 2 School of Graduate Studies, Management and Science University, Shah Alam, Malaysia; 3 Department of Infection, Nanchong Central Hospital, Nanchong, China; 4 North Sichuan Medical College, Nanchong, China

**Keywords:** smoking, laryngeal cancer, Mendelian randomization, causal genetic association

## Abstract

**INTRODUCTION:**

Smoking is well-established as the primary risk factor for laryngeal cancer, yet high-quality clinical randomized controlled trials are lacking. To address this gap, we utilized Mendelian randomization (MR), a novel research approach that offers an alternative to traditional randomized controlled trials. Our study aimed to reaffirm the connection between smoking and laryngeal cancer, while also contributing new insights for global public health prevention.

**METHODS:**

We performed a two-sample MR analysis using publicly released genome-wide association studies (GWAS) statistics. Smoking as exposure and laryngeal cancer as outcome. The inverse-variance weighted (IVW) method was used to analyze the genetic causal association between smoking and laryngeal cancer. We applied four complementary methods, including weighted median, weighted mode, MR-Egger regression, and MR pleiotropy residual sum and outlier (MR-PRESSO) to detect and correct for the effect of horizontal pleiotropy.

**RESULTS:**

Based on IVW, we found a causal association between smoking (cigarettes per day) and laryngeal cancer (OR=9.55; 95% CI: 1.26–72.27; p=0.03). There was a potential genetic causal association between smoking and laryngeal cancer. No heterogeneity (Q=34.06, p=0.89) or horizontal pleiotropy (Egger intercept, p=0.69) was found in any of the analyses. Sensitivity analyses confirmed robustness (MR-PRESSO global test, p=0.96). None of the leave-one-out tests in the analyses found any SNP that could affect the results of MR.

**CONCLUSIONS:**

Genetic liability to smoking is associated with a higher risk of laryngeal cancer. Our findings support a genetic link between smoking and laryngeal cancer, underscoring the importance of smoking prevention in public health strategies.

## INTRODUCTION

Laryngeal cancer is a disease in which malignant cancer cells invade the tissues of the larynx. It ranks second in terms of the occurrence of malignant tumors in the head and neck, constituting approximately 1–5% of all systemic cancers seen in otolaryngology^1^. In 2020, 184615 new cases of laryngeal cancer were diagnosed, and 99840 related deaths were recorded worldwide^2^. The use of tobacco products significantly affects the risk of laryngeal cancer^3^. A substantial body of epidemiological research and case-control studies has consistently demonstrated that smoking is the primary risk factor for laryngeal cancer^3,4^. Previous research has revealed significant correlations between laryngeal cancer and smoking quantity, age of initiation, and cessation^3,5-7^.

Smoking is the single largest preventable cause of illness and death worldwide^8^. Every year, more than 8 million people die from tobacco use^9^. Tobacco can also be deadly for non-smokers, as secondhand smoke exposure has been implicated in adverse health outcomes, causing 1.3 million deaths annually^9^. Human smoking behavior dates back to 5000 BC, originating in the Americas; European engagement commenced in 1492^10^. Presently, approximately 1.3 billion individuals worldwide smoke^9^, representing a decline in prevalence from the previous decade. Cigarette smoke is an exceedingly complex mixture that contains more than 5300 compounds^3^, including multiple toxicants and carcinogens. Smoking is known to severely harm human health, shorten the lifespan, and be associated with the occurrence of more than 20 different types and subtypes of cancer^9^, including laryngeal cancer, lung cancer, and cancer of the lower urinary tract^3^. Laryngeal cancer is among the cancers associated most strongly with cigarette smoking^3^. The International Agency for Research on Cancer has determined that sufficient evidence exists for the carcinogenicity of more than 70 components of tobacco smoke in laboratory animals or humans^3^.

Mendelian randomization (MR) is a technique in which genetic data are used to assess and estimate causal effects of modifiable (non-genetic) risk factors based on observational data^11^. It is an epidemiological method where genetic variants serve as instrumental variables to enhance causal inference^11^. This approach reduces confounding effects and mitigates reverse causality^12,13^. Genetic variants, being allocated randomly at conception, are independent of self-adopted behaviors and environmental factors and remain unaltered by disease onset and progression^11,13,14^.

While a substantial body of basic and clinical research has established smoking as the primary risk factor for laryngeal cancer, yet high-quality clinical randomized controlled trials (RCTs) remain lacking. RCTs are the gold standard for the empirical testing of a scientific hypothesis in a clinical setting^11^, and this research gap potentially raises confounding-factor and reverse-causality issues. To address these limitations effectively, we used MR to reaffirm the longstanding conclusion regarding the association of smoking with laryngeal cancer and to provide new ideas for global public health prevention and control.

## METHODS

### Study design

**Figure 1:**
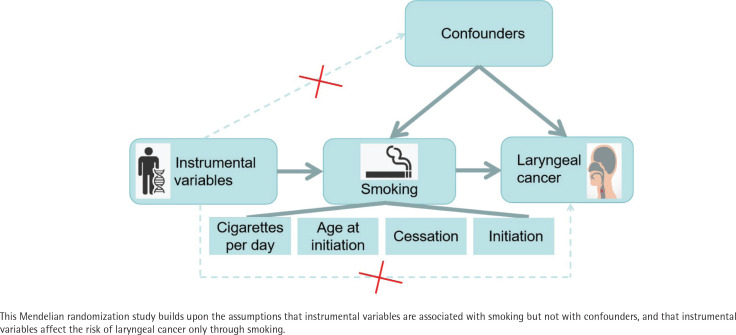
Design of Mendelian randomization study of smoking and risk of laryngeal cancer

The present study was conducted to examine the causal impact of smoking on the risk of laryngeal cancer using a two-sample MR analysis. Individuals’ smoking habits were characterized according to the amount (number of cigarettes/day), history (initiation), age of initiation, and cessation as exposure factors. Laryngeal cancer was considered to be a measure of the resulting outcomes. We ensured that three essential conditions for MR studies were fulfilled: the selected single-nucleotide polymorphisms (SNPs) correlated significantly with the exposure variable, were unrelated to any potential confounding factor, and were related solely to the risk of laryngeal cancer development as a result of smoking. We used aggregated information from published research that had been performed with participant consent and ethical clearance. The study design is summarized in Figure 1.

### Data sources

Meta-analysis results for summary GWAS data from 5053331 European-ancestry individuals were used in this study^15^. Exposure data were obtained from the publicly available GSCAN database library (https://genome.psych.umn.edu/index.php/GSCAN ). The summary data were derived from 59 cohort studies including up to 326497 patients and are presented in the supplementary material (see full statistical outputs in Supplementary file Table S1). Data on smoking initiation (whether an individual ever smoked regularly) were available for 2669029 individuals, those for the age at which individuals began smoking regularly were available for 618514 individuals, those on the amount smoked were available for 618489 current and former regular smokers, and data on smoking cessation (distinguishing current from former smokers)^15^ were available for 1147272 individuals.

Data from GWASs of laryngeal cancer were acquired from the GWAS explorer of the National Cancer Institute (https://exploregwas.cancer.gov/plco-atlas/#/)^16^. Participants in these studies had confirmed diagnoses of cancer of the larynx (International Classification of Diseases for Oncology, second edition, sites C32.0–32.9, morphology excluding hematopoietic cancers, mesothelioma, and Kaposi’s sarcoma). The control group consisted of healthy individuals aged 55–74 years. Summary statistical data were available for up to 28243 individuals (8813042 variants of 89 cases and 28154 controls) of European ancestry. As the exposure data were published prior to 2022 and the outcome data were published in 2023, the sample populations did not overlap.

### Instrumental variable selection

Genetic variants associated with smoking at a genome-wide significance level of p<5×10^-8^ were selected as instruments. Independent SNPs served as instrumental variables (IVs) to prevent counterbalancing resulting from linkage disequilibrium (r^2^ <0.001, clumping window=10000 kb)^17^. SNPs linked to possible confounders [alcohol consumption, inhalation of asbestos and mustard gas, radiation exposure, sex, Zn and Se deficiencies, pharyngolaryngeal reflux, chronic disease, human papillomavirus, and herpes simplex virus (http://www.phenoscanner.medschl.cam.ac.uk/)] were eliminated^18-20^. To rectify allele orientation, SNP harmonization was performed. The F statistic was used as a supplementary assessment of instrumental variable strength. The following equation was used: F=R^2^(N-K-1)/[K(1-R^2^)], where R^2^ is the total accounted variance of the selected SNP throughout exposure, N is the sample size of the exposed database, and K is the number of SNPs included in the final analysis. The F statistics for all instrument–exposure effects exceeded the recommended threshold for MR analyses of F>10 (see full statistical outputs in Supplementary file Table S1), reflecting a low likelihood of weak instrumental bias^21^.

### Statistical analysis

The MR analyses were performed using the Two Sample MR^22^, Mendelian randomization^23^, and MR-PRESSO^24^ packages in R (version 4.3.1). The primary MR analysis was performed using the inverse-variance weighted (IVW) method^14^. Cochran’s Q test was used to assess heterogeneity among assessments of particular genetic variations^25^. In addition to the IVW method^24,26^, the maximum likelihood, weighted median, and MR-Egger regression methods were used. Scatter plots of associations between genetically determined smoking and laryngeal cancer results were generated. To validate the IVW findings, the MR-PRESSO package was used to examine and calibrate horizontal pleiotropic outliers. When only the SNP ID number was missing, genetic locus information was used to find the SNP number and complete the data; otherwise, the SNP was excluded.

### Sensitivity analysis

To identify potential pleiotropy, the MR-Egger test was conducted; intercept p>0.05 were taken to indicate the absence of horizontal pleiotropy^27^. To evaluate the robustness of the findings, leave-one-out sensitivity analyses (involving the systematic exclusion of one SNP at a time) were performed^28^. To directly investigate the presence of pleiotropy, forest, and funnel plots were created^29^.

The reporting of this study adheres to the STROBE-MR checklist. Publicly accessible data were used, and the individual studies from which they were derived were approved by the appropriate institutional review boards and performed with participants’ or authorized representatives’ informed consent.

## RESULTS

### Instrumental variables

Information on the smoking-related instrumental variables included in the MR analysis is provided in supplementary material (see full statistical outputs in Supplementary file Table S2). For more detailed phenotypic information, please refer to Supplementary file Table S3. These variables comprised 46 single-nucleotide polymorphisms (SNPs) related to the smoking amount, 226 related to smoking initiation, 10 related to the age at regular smoking initiation, and 20 SNPs related to smoking cessation. Detailed information on SNP inclusion and exclusion at each stage is provided in the supplementary material (as show in Supplementary file Figure S1).

### Genetically determined smoking phenotypes and laryngeal cancer

The conventional IVW approach revealed that a genetic predisposition toward higher smoking amount was associated with an elevated likelihood of laryngeal cancer development (OR=9.55; 95% CI: 1.27–72.27; p=0.03). Genetically predicted smoking initiation, age of smoking initiation, and smoking cessation were not associated with laryngeal cancer development (Table 1).

**Table 1:** Mendelian randomization for the examination of the effect of smoking on the risk of laryngeal cancer in European-ancestry individuals

*Exposure*	*Method*	*SNP* *n*	*β*	*p*	*OR*	*95% CI*	
						*Lower*	*Upper*
Smoking amount (cigarettes/day)	IVW	46	2.26	0.03*	9.55	1.27	72.27
Smoking initiation	IVW	226	0.61	0.53	1.83	0.28	12.01
Age at smoking initiation	IVW	10	1.97	0.54	7.02	0.02	3331.66
Smoking cessation	IVW	20	-1.06	0.64	0.35	0.01	24.71

IVW: inverse-variance weighted. SNP: single-nucleotide polymorphism.

* Statistical significance threshold: p<0.05 for all tests. Data sources: Exposure variables from GSCAN consortium, 2022 (N=326497); Outcome from NCI GWAS Explorer, 2023 (N=28243).

### Sensitivity analysis results

**Figure 2:**
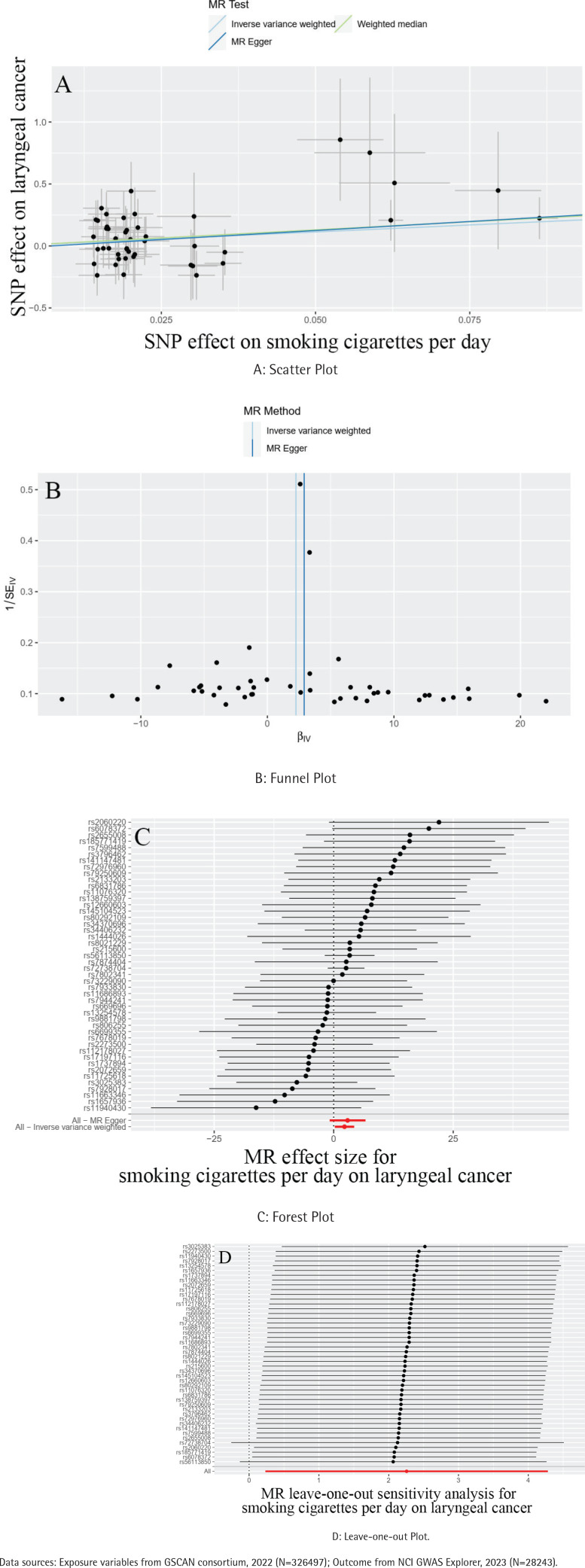
Mendelian randomization for smoking cigarettes per day on the risk of laryngeal cancer. A: Scatter Plot, B: Funnel Plot, C: Forest Plot, and D: Leave-one-out Plot.

The MR-Egger intercepts showed no evidence of directional pleiotropy (all p>0.05), indicating that the observed associations were not likely due to confounding by pleiotropic effects. The causal estimates obtained through MR-PRESSO analysis remained consistent before and after correction for outliers, supporting the robustness of the MR results (Table 2). Although the heterogeneity test suggested potential variations in outcomes, the Cochran Q test revealed no significant difference among the SNPs related to laryngeal cancer (Table 2). Additional analyses, including scatter, funnel, and forest plots of the relationship between smoking amount and laryngeal cancer development, are provided in Figures 2A–2C. The leave-one-out analysis indicated that no specific genetic variation significantly impacted the overall estimation of causality (Figure 2D).

**Table 2:** Pleiotropy and heterogeneity tests for the effect of smoking on the risk of laryngeal cancer in European-ancestry individuals

*Exposure*	*Pleiotropy test*				*Heterogeneity test*					
	*MR-Egger*			*MR-PRESSO*	*MR-Egger*			*Inverse-variance weighted*		
	*Intercept*	*SE*	*p*	*p*	*Q*	*Q_df*	*Q_p*	*Q*	*Q_df*	*Q_p*
Smoking amount (cigarettes/day)	-0.02	0.05	0.69	0.96	33.90	44	0.86	34.06	45	0.88
Smoking initiation	0.08	0.05	0.09	0.96	193.96	224	0.93	196.86	225	0.91
Age at smoking initiation	0.32	0.26	0.26	0.86	3.06	8	0.93	4.56	9	0.87
Smoking cessation	0.02	0.12	0.87	0.30	22.02	18	0.23	22.05	19	0.28

## DISCUSSION

This two-sample MR analysis revealed that genetically predicted smoking is associated with an increased likelihood of developing laryngeal cancer in the European population. However, smoking initiation, age of smoking initiation, and smoking cessation were not related to the laryngeal cancer risk in this population.

Clinical observational studies have consistently revealed strong associations between laryngeal cancer and smoking, particularly long-term and heavy smoking, but whether smoking is the primary etiological factor for laryngeal cancer remains incompletely understood^30^. To our knowledge, this study is the first in which MR analysis was used to investigate the relationship between extensive smoking and laryngeal cancer. MR complements traditional epidemiological methods, as genetic variants are used as IVs to estimate causal effects and reverse causality and the effects of confounders (e.g. sex and alcohol use) are avoided. The observed association aligns with the observational findings.

A comprehensive review of the existing literature suggests that smoking contributes to laryngeal cancer through four potential mechanisms. First, upon the inhalation of tobacco smoke, larger particles are deposited primarily in the laryngeal mucosa. Secondary flows generated by turbulence due to the narrowed cross-sectional area and the complex topographic structure of the human larynx lead to the deposition of fine and ultrafine particles. Additional deposition, especially of fine and ultrafine particles, occurs during smoke exhalation. The increased accumulation of tobacco smoke in the laryngeal area increases the susceptibility to cancer relative to other portions of the respiratory tract^31^. Second, local inflammation caused by specific constituents of tobacco smoke has been implicated. Many patients with laryngeal cancer exhibit chronic laryngeal inflammation^32^. Third, polycyclic aromatic hydrocarbons and nitrosamines are the primary cancer-causing agents in smoke. Enzymes such as aryl hydrocarbon hydroxylase break these hydrocarbons down into cancer-causing substances. The genetically determined enzymes contribute to the variations observed in individuals’ susceptibility to the carcinogenic effects of smoking. Fourth, cigarette smoke triggers the activation of pulmonary alveolar macrophages, leading to superoxide and hydrogen peroxide production and thereby contributing to the oxidative damage of DNA and RNA, increasing the likelihood of carcinogenesis^33^.

Individuals who initiated smoking before the age of 20 years have been found to be most susceptible to laryngeal cancer development, whereas smoking initiation at older ages has been linked to a lower risk than observed in a control group. In the present study based on genetic prediction, no causal relationship was observed between smoking initiation or the age thereof and laryngeal cancer. Participants in the previous study had continued smoking without cessation after initiation^29^, whereas the population in which the age of smoking initiation was examined in this study included current and former smokers. This discrepancy may contribute to the difference in outcomes.

Some study results suggest that smoking cessation reduces the risk of laryngeal cancer^18,34,35^, whereas the present study revealed no genetic correlation. In agreement with our findings, a meta-analysis showed that the laryngeal cancer risk remained elevated for 15 years after smoking cessation^5^. People who have quit smoking may have smoked more than those who have not quit, which may explain our findings.

### Strengths and limitations

Our investigation has specific strengths. First, the outcome and exposure data were derived from separate samples, which bolstered the statistical power to discern subtle influences on complex characteristics. It also increased the total sample size and thus the precision of causal effect estimation. Additionally, rigorous standards were applied to IV selection to ensure that only smoking-related variants that correlated significantly with smoking measures and conformed to the three fundamental premises of MR analysis were chosen. Furthermore, the genetic variants were located on separate chromosomes, suggesting that potential interplay between genes had minimal influence on the estimations.

Our study has some limitations that should be acknowledged. First, heterogeneity may have affected the analysis. As we relied on GWAS data, we were unable to explore potential nonlinear relationships or stratification effects (e.g. differences according to health status and age), which could have contributed to heterogeneity. Second, although efforts were made to account for potential pleiotropy, its presence could not be excluded definitively because it could introduce distortion in causal effect estimation. Third, although we specifically removed SNPs linked to recognized confounding factors, additional uncharted confounders could exist and could have affected the observed association between smoking and laryngeal cancer; further investigation is warranted to explore this possibility. Fourth, the populations examined were exclusively of European ancestry, limiting the generalizability of our findings due to the potential for variation in disease patterns among populations with different backgrounds. Finally, although the IVW method indicated a significant causal effect of smoking quantity on laryngeal cancer (OR=9.55, p=0.03), the extremely wide confidence interval (95% CI: 1.26–72.27) warrants cautious interpretation. This imprecision primarily stems from the limited statistical power of the outcome dataset, which included only 89 laryngeal cancer cases. Small outcome sample sizes reduce the accuracy of genetic association estimates for rare variants, amplifying standard errors and resulting in unstable effect sizes. While the point estimate (OR=9.55) aligns with epidemiological evidence demonstrating strong smoking–laryngeal cancer associations, the broad CI indicates that the true effect could range from marginal to exceedingly high. Additional research is needed to confirm the impact of smoking on laryngeal cancer, including comprehensive RCTs and large-sample MR studies to validate our MR findings. Phenotypic details were inaccessible from the source GWAS summary statistics. While this does not affect genetic instrument validity, it precludes subgroup analyses. Future studies with individual-level data could address this gap.

## CONCLUSIONS

This two-sample MR study yielded genetic evidence suggesting that smoking increases the likelihood of laryngeal cancer development. No causal link was established between smoking initiation, the age of smoking initiation, or smoking cessation and the risk of laryngeal cancer; however, we still recommend the avoidance of smoking and, for current smokers, its cessation as early as possible.

## Supplementary Material



## Data Availability

The data supporting this research are available from the following sources: https://genome.psych.umn.edu/index.php/GSCAN, https://exploregwas.cancer.gov/plco-atlas/#/
