# Supplementary material for: Causal association of smoking and laryngeal cancer: A Mendelian randomization study
Source: Tob Induc Dis. 2025 Nov 21;23:10.18332/tid/209744. doi: 10.18332/tid/209744 (PMC12639414; doi:10.18332/tid/209744)
Supplement: Supplementary file 1 [file TID-23-180-s1.pdf]

Table S1. SNP information and F values.

Age at smoking initiation

	SNP	effect_allele	other_allele	effect_allele	other_allele	beta.exposu	beta.outcor	eaf.exposur	eaf.outcom	remove	palindromic	ambiguous	id.outcome	se.outcome	pval.outcon	outcome	mr_keep.ou	pval_origin.	data_source
1	rs11080208	C	T	C	T	-0.0176	-0.1414	0.752485	0.75	FALSE	FALSE	FALSE	Dbvu5u	0.1846	0.4436	outcome	TRUE	reported	textfile
2	rs11780471	A	G	A	G	0.0369	-0.3499	0.0755467	0.08	FALSE	FALSE	FALSE	Dbvu5u	0.3151	0.2668	outcome	TRUE	reported	textfile
3	rs11915747	G	C	G	C	0.0174	0.1286	0.367793	0.37	FALSE	TRUE	FALSE	Dbvu5u	0.1622	0.4279	outcome	TRUE	reported	textfile
4	rs12575642	T	G	T	G	-0.0183	-0.1839	0.183897	0.18	FALSE	FALSE	FALSE	Dbvu5u	0.1988	0.3548	outcome	TRUE	reported	textfile
5	rs241979	A	G	A	G	-0.0159	-0.0101	0.246521	0.25	FALSE	FALSE	FALSE	Dbvu5u	0.1809	0.9557	outcome	TRUE	reported	textfile
6	rs2491383	G	T	G	T	0.0152	-0.1024	0.61829	0.62	FALSE	FALSE	FALSE	Dbvu5u	0.1584	0.5181	outcome	TRUE	reported	textfile
7	rs2607015	C	G	C	G	0.0154	-0.1235	0.441352	0.37	FALSE	TRUE	TRUE	Dbvu5u	0.1694	0.466	outcome	TRUE	reported	textfile
8	rs4692334	C	G	C	G	0.018	0.1667	0.803181	0.8	FALSE	TRUE	FALSE	Dbvu5u	0.2016	0.4083	outcome	TRUE	reported	textfile
9	rs571312	A	C	A	C	0.0176	0.039	0.239563	0.24	FALSE	FALSE	FALSE	Dbvu5u	0.1817	0.8299	outcome	TRUE	reported	textfile
10	rs62180314	C	G	C	G	0.0236	-0.0282	0.207753	0.21	FALSE	TRUE	FALSE	Dbvu5u	0.1914	0.8828	outcome	TRUE	reported	textfile
11	rs624833	G	T	G	T	0.0158	0.115	0.328032	0.33	FALSE	FALSE	FALSE	Dbvu5u	0.1678	0.493	outcome	TRUE	reported	textfile

	SNP	X	chr.exposur	pos.exposu	se.exposure	pval.exposu	ncase.expos	exposure	mr_keep.ex	pval_origin.	id.exposure	data_source	action	mr_keep	samplesize.	EAF2	MAF	PVE	FSTAT
1	rs11080208	60	17	33223425	0.003	8.63E-10	323386	exposure	TRUE	reported	8UeI21	textfile	2	TRUE	NA	0.247515	0.247515	0.0001064	34.417565
2	rs11780471	681	8	27487202	0.005	1.59E-12	323386	exposure	TRUE	reported	8UeI21	textfile	2	TRUE	NA	0.9244533	0.0755467	0.0001684	54.464063
3	rs11915747	626	3	85649890	0.003	1.75E-11	323386	exposure	TRUE	reported	8UeI21	textfile	2	TRUE	NA	0.632207	0.367793	0.000104	33.639792
4	rs12575642	26	11	64212171	0.003	1.69E-08	323386	exposure	TRUE	reported	8UeI21	textfile	2	TRUE	NA	0.816103	0.183897	0.0001151	37.20977
5	rs241979	27	12	3725308	0.003	3.87E-08	323386	exposure	TRUE	reported	8UeI21	textfile	2	TRUE	NA	0.753479	0.246521	8.69E-05	28.089826
6	rs2491383	2	10	104856882	0.003	1.30E-08	290963	exposure	TRUE	reported	8UeI21	textfile	2	TRUE	NA	0.38171	0.38171	8.82E-05	25.670935
7	rs2607015	677	6	31795066	0.003	9.60E-09	288075	exposure	TRUE	reported	8UeI21	textfile	2	FALSE	NA	0.558648	0.441352	9.15E-05	26.350928
8	rs4692334	666	4	28615391	0.003	4.05E-08	323386	exposure	TRUE	reported	8UeI21	textfile	2	TRUE	NA	0.196819	0.196819	0.0001113	35.999777
9	rs571312	90	18	60172536	0.003	2.21E-09	323386	exposure	TRUE	reported	8UeI21	textfile	2	TRUE	NA	0.760437	0.239563	0.0001064	34.417565
10	rs62180314	151	2	63145998	0.003	1.39E-14	323386	exposure	TRUE	reported	8UeI21	textfile	2	TRUE	NA	0.792247	0.207753	0.0001913	61.884062
11	rs624833	670	4	2879529	0.003	4.98E-09	323386	exposure	TRUE	reported	8UeI21	textfile	2	TRUE	NA	0.671968	0.328032	8.58E-05	27.737606

Smoking cessation

	SNP	effect_allel	other_allele	effect_allel	other_allele	beta.exposu	beta.outcor	eaf.exposur	eaf.outcom	remove	palindromic	ambiguous
1	rs1009181	C	T	C	T	-0.013	-0.1718	0.33499	0.33	FALSE	FALSE	FALSE
2	rs10182249	A	G	A	G	-0.0136	0.2413	0.646123	0.65	FALSE	FALSE	FALSE
3	rs10402271	G	T	G	T	-0.0132	0.3054	0.34493	0.34	FALSE	FALSE	FALSE
4	rs10821537	G	A	G	A	-0.0153	0.1648	0.652087	0.63	FALSE	FALSE	FALSE
5	rs11338241	A	C	A	C	0.0501	-0.3238	0.0904573	0.09	FALSE	FALSE	FALSE
6	rs11697662	T	C	T	C	-0.0301	0.0315	0.804175	0.79	FALSE	FALSE	FALSE
7	rs11991338	A	G	A	G	0.0182	0.1426	0.170974	0.17	FALSE	FALSE	FALSE
8	rs2006281	T	C	T	C	-0.0139	-0.1556	0.534791	0.53	FALSE	FALSE	FALSE
9	rs2118362	C	T	C	T	0.0153	-0.064	0.286282	0.28	FALSE	FALSE	FALSE
10	rs215600	A	G	A	G	-0.0144	-0.0754	0.667992	0.66	FALSE	FALSE	FALSE
11	rs35033100	C	T	C	T	-0.0307	-0.4646	0.0417495	0.04	FALSE	FALSE	FALSE
12	rs4705014	A	G	A	G	-0.0132	0.1147	0.661034	0.66	FALSE	FALSE	FALSE
13	rs56049603	G	C	G	C	0.0159	-0.2273	0.202783	0.2	FALSE	TRUE	FALSE
14	rs56113850	C	T	C	T	-0.033	0.2074	0.592445	0.58	FALSE	FALSE	FALSE
15	rs591143	T	C	T	C	-0.0144	-0.0684	0.629225	0.63	FALSE	FALSE	FALSE
16	rs60749569	T	A	T	A	-0.0266	-0.2094	0.0805169	0.08	FALSE	TRUE	FALSE
17	rs71563618	G	A	G	A	0.0207	0.4042	0.084493	0.09	FALSE	FALSE	FALSE
18	rs72740955	T	C	T	C	0.0252	0.1454	0.373757	0.37	FALSE	FALSE	FALSE
19	rs7807019	G	A	G	A	0.0147	-0.0912	0.470179	0.47	FALSE	FALSE	FALSE
20	rs9607805	T	C	T	C	0.0189	0.2515	0.702783	0.7	FALSE	FALSE	FALSE

SNP	id.outcome	se.outcome	pval.outcon	outcome	mr_keep.ou	pval_origin.	data_source	X	chr.exposur	pos.exposu	se.exposure	pval.exposu
rs1009181	E9Z4TX	0.1681	0.3068	outcome	TRUE	reported	textfile	1885	6	26158765	0.002	3.71E-08
rs10182249	E9Z4TX	0.1656	0.1451	outcome	TRUE	reported	textfile	1247	2	22741564	0.002	1.61E-08
rs10402271	E9Z4TX	0.1641	0.06263	outcome	TRUE	reported	textfile	765	19	44825957	0.002	4.30E-08
rs10821537	E9Z4TX	0.1769	0.3515	outcome	TRUE	reported	textfile	2235	9	133621702	0.002	1.58E-10
rs11338241	E9Z4TX	0.2522	0.1992	outcome	TRUE	reported	textfile	2170	9	133597897	0.004	4.96E-41
rs11697662	E9Z4TX	0.2001	0.8751	outcome	TRUE	reported	textfile	855	20	63360653	0.003	2.26E-25
rs11991338	E9Z4TX	0.2471	0.5639	outcome	TRUE	reported	textfile	2024	8	9430954	0.003	2.31E-08
rs2006281	E9Z4TX	0.1569	0.3214	outcome	TRUE	reported	textfile	61	14	103861395	0.002	8.11E-10
rs2118362	E9Z4TX	0.1786	0.7203	outcome	TRUE	reported	textfile	3	11	16351537	0.003	2.19E-09
rs215600	E9Z4TX	0.1615	0.6405	outcome	TRUE	reported	textfile	2008	7	32294030	0.002	1.50E-09
rs35033100	E9Z4TX	0.3868	0.2297	outcome	TRUE	reported	textfile	767	20	63095021	0.005	2.31E-08
rs4705014	E9Z4TX	0.1621	0.4793	outcome	TRUE	reported	textfile	1884	5	156425305	0.002	1.98E-08
rs56049603	E9Z4TX	0.1832	0.2149	outcome	TRUE	reported	textfile	1423	3	49547810	0.003	8.42E-09
rs56113850	E9Z4TX	0.165	0.2087	outcome	TRUE	reported	textfile	658	19	40847202	0.002	3.85E-47
rs591143	E9Z4TX	0.1571	0.6635	outcome	TRUE	reported	textfile	169	15	47355558	0.002	5.00E-10
rs60749569	E9Z4TX	0.2878	0.4668	outcome	TRUE	reported	textfile	2023	8	42747525	0.004	5.38E-10
rs71563618	E9Z4TX	0.2526	0.1096	outcome	TRUE	reported	textfile	1886	7	115197750	0.004	6.91E-09
rs72740955	E9Z4TX	0.1653	0.3792	outcome	TRUE	reported	textfile	345	15	78557437	0.002	1.30E-25
rs7807019	E9Z4TX	0.1564	0.5598	outcome	TRUE	reported	textfile	1915	7	117903009	0.002	1.00E-10
rs9607805	E9Z4TX	0.1764	0.1539	outcome	TRUE	reported	textfile	1172	22	41458442	0.003	8.88E-14

SNP	ncase.expo	exposure	mr_keep.ex	pval_origin.	id.exposure	data_source	action	mr_keep	samplesize.	EAF2	MAF	PVE	FSTAT
rs1009181	388313	exposure	TRUE	reported	FYQyoV	textfile	2	TRUE	NA	0.66501	0.33499	0.0001088	42.249782
rs10182249	388313	exposure	TRUE	reported	FYQyoV	textfile	2	TRUE	NA	0.353877	0.353877	0.0001191	46.239762
rs10402271	388313	exposure	TRUE	reported	FYQyoV	textfile	2	TRUE	NA	0.65507	0.34493	0.0001122	43.559776
rs10821537	375952	exposure	TRUE	reported	FYQyoV	textfile	2	TRUE	NA	0.347913	0.347913	0.0001556	58.522189
rs11338241	388313	exposure	TRUE	reported	FYQyoV	textfile	2	TRUE	NA	0.9095427	0.0904573	0.0004038	156.87482
rs11697662	378958	exposure	TRUE	reported	FYQyoV	textfile	2	TRUE	NA	0.195825	0.195825	0.0002656	100.66725
rs11991338	333791	exposure	TRUE	reported	FYQyoV	textfile	2	TRUE	NA	0.829026	0.170974	0.0001102	36.804224
rs2006281	388313	exposure	TRUE	reported	FYQyoV	textfile	2	TRUE	NA	0.465209	0.465209	0.0001244	48.302251
rs2118362	388313	exposure	TRUE	reported	FYQyoV	textfile	2	TRUE	NA	0.713718	0.286282	6.70E-05	26.009866
rs215600	388313	exposure	TRUE	reported	FYQyoV	textfile	2	TRUE	NA	0.332008	0.332008	0.0001335	51.839733
rs35033100	378958	exposure	TRUE	reported	FYQyoV	textfile	2	TRUE	NA	0.9582505	0.0417495	9.95E-05	37.699401
rs4705014	388313	exposure	TRUE	reported	FYQyoV	textfile	2	TRUE	NA	0.338966	0.338966	0.0001122	43.559776
rs56049603	388313	exposure	TRUE	reported	FYQyoV	textfile	2	TRUE	NA	0.797217	0.202783	7.23E-05	28.089855
rs56113850	388313	exposure	TRUE	reported	FYQyoV	textfile	2	TRUE	NA	0.407555	0.407555	0.0007006	272.2486
rs591143	388312	exposure	TRUE	reported	FYQyoV	textfile	2	TRUE	NA	0.370775	0.370775	0.0001335	51.839733
rs60749569	355742	exposure	TRUE	reported	FYQyoV	textfile	2	TRUE	NA	0.9194831	0.0805169	0.0001243	44.222251
rs71563618	388313	exposure	TRUE	reported	FYQyoV	textfile	2	TRUE	NA	0.915507	0.084493	6.90E-05	26.780487
rs72740955	388312	exposure	TRUE	reported	FYQyoV	textfile	2	TRUE	NA	0.626243	0.373757	0.0004087	158.75918
rs7807019	388313	exposure	TRUE	reported	FYQyoV	textfile	2	TRUE	NA	0.529821	0.470179	0.0001391	54.022222
rs9607805	388313	exposure	TRUE	reported	FYQyoV	textfile	2	TRUE	NA	0.297217	0.297217	0.0001022	39.689796

Smoking cigarettes per day

	SNP	effect_allel	other_allel	effect_allel	other_allel	beta.exposu	beta.outcor	eaf.exposur	eaf.outcom	remove	palindromic	ambiguous	id.outcome	se.outcome	pval.outcon	outcome	mr_keep.ou	pval_origin.	data_source	X
1	rs11076320	A	C	A	C	-0.0164	-0.138	0.632207	0.63	FALSE	FALSE	FALSE	Z1glEx	0.1628	0.3966	outcome	TRUE	reported	textfile	1839
2	rs11217802	T	C	T	C	0.0205	-0.0866	0.181909	0.18	FALSE	FALSE	FALSE	Z1glEx	0.2111	0.6816	outcome	TRUE	reported	textfile	2096
3	rs11663346	T	A	T	A	-0.0141	0.1448	0.592445	0.59	FALSE	TRUE	FALSE	Z1glEx	0.1582	0.3599	outcome	TRUE	reported	textfile	2116
4	rs11686893	C	T	C	T	0.0165	-0.0193	0.286282	0.29	FALSE	FALSE	FALSE	Z1glEx	0.1668	0.9078	outcome	TRUE	reported	textfile	3443
5	rs11725618	C	T	C	T	0.0181	-0.1053	0.313121	0.31	FALSE	FALSE	FALSE	Z1glEx	0.1713	0.5387	outcome	TRUE	reported	textfile	3824
6	rs11940430	A	T	A	T	-0.0146	0.2369	0.654076	0.65	FALSE	TRUE	FALSE	Z1glEx	0.1638	0.1483	outcome	TRUE	reported	textfile	3954
7	rs12660603	C	T	C	T	-0.0303	-0.2385	0.0437376	0.04	FALSE	FALSE	FALSE	Z1glEx	0.3531	0.4995	outcome	TRUE	reported	textfile	4024
8	rs13254578	C	G	C	G	0.0353	-0.0505	0.777336	0.77	FALSE	TRUE	FALSE	Z1glEx	0.1854	0.7853	outcome	TRUE	reported	textfile	4283
9	rs13875939	C	G	C	G	0.0628	0.5082	0.0258449	0.03	FALSE	TRUE	FALSE	Z1glEx	0.5573	0.3618	outcome	TRUE	reported	textfile	1170
10	rs14114748	G	C	G	C	-0.0588	-0.7523	0.0228628	0.02	FALSE	TRUE	FALSE	Z1glEx	0.6063	0.2147	outcome	TRUE	reported	textfile	775
11	rs1444026	G	T	G	T	0.014	0.0741	0.446322	0.44	FALSE	FALSE	FALSE	Z1glEx	0.1669	0.6573	outcome	TRUE	reported	textfile	4165
12	rs14510452	T	C	T	C	0.0212	0.1482	0.125249	0.12	FALSE	FALSE	FALSE	Z1glEx	0.2323	0.5236	outcome	TRUE	reported	textfile	3436
13	rs1657936	T	C	T	C	-0.0189	0.2317	0.781312	0.78	FALSE	FALSE	FALSE	Z1glEx	0.1977	0.2411	outcome	TRUE	reported	textfile	473
14	rs17197116	C	T	C	T	0.0298	-0.154	0.084493	0.08	FALSE	FALSE	FALSE	Z1glEx	0.2852	0.5892	outcome	TRUE	reported	textfile	176
15	rs1737894	G	C	G	C	0.0192	-0.1008	0.377734	0.37	FALSE	TRUE	FALSE	Z1glEx	0.1659	0.5433	outcome	TRUE	reported	textfile	3308
16	rs18577141	T	G	T	G	-0.054	-0.857	0.0238569	0.03	FALSE	FALSE	FALSE	Z1glEx	0.4931	0.08221	outcome	TRUE	reported	textfile	1625
17	rs2016968	G	C	G	C	-0.0193	0.0185	0.571571	0.57	FALSE	TRUE	TRUE	Z1glEx	0.1594	0.9076	outcome	TRUE	reported	textfile	1978
18	rs2060220	T	A	T	A	0.0201	0.4426	0.133201	0.13	FALSE	TRUE	FALSE	Z1glEx	0.2358	0.06047	outcome	TRUE	reported	textfile	3955
19	rs2072659	G	C	G	C	-0.0301	0.1616	0.101392	0.09	FALSE	TRUE	FALSE	Z1glEx	0.2668	0.5447	outcome	TRUE	reported	textfile	3029
20	rs2133203	T	C	T	C	-0.0162	-0.1546	0.450298	0.45	FALSE	FALSE	FALSE	Z1glEx	0.1572	0.3256	outcome	TRUE	reported	textfile	3065
21	rs215600	A	G	A	G	-0.0225	-0.0754	0.667992	0.66	FALSE	FALSE	FALSE	Z1glEx	0.1615	0.6405	outcome	TRUE	reported	textfile	4111
22	rs2273500	C	T	C	T	0.035	-0.1402	0.146123	0.14	FALSE	FALSE	FALSE	Z1glEx	0.2175	0.5193	outcome	TRUE	reported	textfile	3405
23	rs2655008	T	A	T	A	0.0161	0.2568	0.730616	0.73	FALSE	TRUE	FALSE	Z1glEx	0.1786	0.1505	outcome	TRUE	reported	textfile	3458
24	rs3025383	C	T	C	T	-0.0307	0.2368	0.180915	0.18	FALSE	FALSE	FALSE	Z1glEx	0.1981	0.232	outcome	TRUE	reported	textfile	4669
25	rs34370696	T	C	T	C	-0.0192	-0.1108	0.175944	0.18	FALSE	FALSE	FALSE	Z1glEx	0.212	0.6011	outcome	TRUE	reported	textfile	3040
26	rs34406232	A	C	A	C	-0.0796	-0.4474	0.0258449	0.02	FALSE	FALSE	FALSE	Z1glEx	0.4742	0.3454	outcome	TRUE	reported	textfile	2523
27	rs3796462	T	C	T	C	-0.0147	-0.2045	0.701789	0.7	FALSE	FALSE	FALSE	Z1glEx	0.1659	0.2176	outcome	TRUE	reported	textfile	3949
28	rs56113850	C	T	C	T	0.0622	0.2074	0.592445	0.58	FALSE	FALSE	FALSE	Z1glEx	0.165	0.2087	outcome	TRUE	reported	textfile	2661
29	rs6078372	A	G	A	G	0.0153	0.3046	0.420477	0.42	FALSE	FALSE	FALSE	Z1glEx	0.1578	0.05355	outcome	TRUE	reported	textfile	3080
30	rs6603895	A	T	A	T	-0.014	-0.0383	0.513916	0.51	FALSE	TRUE	TRUE	Z1glEx	0.1569	0.8071	outcome	TRUE	reported	textfile	3050
31	rs669696	A	C	A	C	-0.0194	0.0257	0.423459	0.42	FALSE	FALSE	FALSE	Z1glEx	0.1556	0.8688	outcome	TRUE	reported	textfile	1882
32	rs6699355	T	C	T	C	-0.0207	0.0679	0.877734	0.85	FALSE	FALSE	FALSE	Z1glEx	0.2625	0.7958	outcome	TRUE	reported	textfile	3060
33	rs6831786	A	C	A	C	-0.0161	-0.1405	0.558648	0.55	FALSE	FALSE	FALSE	Z1glEx	0.1572	0.3715	outcome	TRUE	reported	textfile	3917
34	rs72738704	C	G	C	G	0.0863	0.2238	0.370775	0.37	FALSE	TRUE	FALSE	Z1glEx	0.1689	0.1852	outcome	TRUE	reported	textfile	531
35	rs72976960	G	A	G	A	-0.0207	-0.2577	0.184891	0.18	FALSE	FALSE	FALSE	Z1glEx	0.2144	0.2295	outcome	TRUE	reported	textfile	2232
36	rs73229090	A	C	A	C	0.0304	-0.001	0.11332	0.11	FALSE	FALSE	FALSE	Z1glEx	0.2385	0.9965	outcome	TRUE	reported	textfile	4196
37	rs7599488	T	C	T	C	0.0144	0.2116	0.417495	0.42	FALSE	FALSE	FALSE	Z1glEx	0.1556	0.174	outcome	TRUE	reported	textfile	3456
38	rs7678019	A	G	A	G	-0.018	0.068	0.378728	0.38	FALSE	FALSE	FALSE	Z1glEx	0.1613	0.6733	outcome	TRUE	reported	textfile	3774
39	rs7802341	A	T	A	T	-0.0223	-0.0404	0.746521	0.71	FALSE	TRUE	FALSE	Z1glEx	0.1949	0.8358	outcome	TRUE	reported	textfile	4161
40	rs7874404	T	C	T	C	-0.02	-0.0526	0.213718	0.2	FALSE	FALSE	FALSE	Z1glEx	0.1952	0.7877	outcome	TRUE	reported	textfile	4625
41	rs79250609	T	C	T	C	-0.0189	-0.2268	0.176938	0.17	FALSE	FALSE	FALSE	Z1glEx	0.2155	0.2927	outcome	TRUE	reported	textfile	367
42	rs7928017	A	C	A	C	-0.0176	0.1523	0.407555	0.42	FALSE	FALSE	FALSE	Z1glEx	0.156	0.329	outcome	TRUE	reported	textfile	17
43	rs7933830	T	C	T	C	0.0194	-0.0209	0.319085	0.32	FALSE	FALSE	FALSE	Z1glEx	0.173	0.9039	outcome	TRUE	reported	textfile	99
44	rs7944241	G	A	G	A	-0.0156	0.0195	0.409543	0.41	FALSE	FALSE	FALSE	Z1glEx	0.1582	0.9019	outcome	TRUE	reported	textfile	125
45	rs8021229	T	C	T	C	0.0176	0.0595	0.296223	0.29	FALSE	FALSE	FALSE	Z1glEx	0.1649	0.7183	outcome	TRUE	reported	textfile	414
46	rs80292109	G	A	G	A	-0.0194	-0.1275	0.295229	0.31	FALSE	FALSE	FALSE	Z1glEx	0.1724	0.4593	outcome	TRUE	reported	textfile	3979
47	rs806255	A	G	A	G	-0.0197	0.0452	0.732604	0.73	FALSE	FALSE	FALSE	Z1glEx	0.1773	0.7989	outcome	TRUE	reported	textfile	4530
48	rs9522262	G	C	G	C	0.0144	0.0198	0.519881	0.52	FALSE	TRUE	TRUE	Z1glEx	0.1582	0.9005	outcome	TRUE	reported	textfile	372
49	rs9881798	C	A	C	A	0.0147	-0.0263	0.39165	0.39	FALSE	FALSE	FALSE	Z1glEx	0.1575	0.8674	outcome	TRUE	reported	textfile	3463

	CHR.exposu	pos.exposu	se.exposure	pval.exposu	ncase.expos	exposure	mr_keep.ex	pval_origin.	id.exposure	data_source	action	mr_keep	samplesize.	EAF2	MAF	PVE	FSTAT
1	16	52063640	0.003	1.06E-10	324557	exposure	TRUE	reported	88xvfw	textfile	2	TRUE	NA	0.367793	0.367793	9.21E-05	29.88426
2	17	29236995	0.003	8.02E-10	326497	exposure	TRUE	reported	88xvfw	textfile	2	TRUE	NA	0.818091	0.181909	0.000143	46.694158
3	18	64485223	0.003	1.75E-08	326497	exposure	TRUE	reported	88xvfw	textfile	2	TRUE	NA	0.407555	0.407555	6.77E-05	22.089865
4	2	147737481	0.003	3.28E-10	326497	exposure	TRUE	reported	88xvfw	textfile	2	TRUE	NA	0.713718	0.286282	9.26E-05	30.249815
5	4	66188051	0.003	5.71E-11	326497	exposure	TRUE	reported	88xvfw	textfile	2	TRUE	NA	0.686879	0.313121	0.0001115	36.400888
6	4	98588302	0.003	2.94E-08	326497	exposure	TRUE	reported	88xvfw	textfile	2	TRUE	NA	0.345924	0.345924	7.25E-05	23.684299
7	6	97522873	0.006	4.24E-08	300003	exposure	TRUE	reported	88xvfw	textfile	2	TRUE	NA	0.9562624	0.0437376	8.50E-05	25.50233
8	8	42690703	0.003	4.81E-33	326497	exposure	TRUE	reported	88xvfw	textfile	2	TRUE	NA	0.222664	0.222664	0.0004239	138.4536
9	15	78719146	0.009	5.71E-13	325689	exposure	TRUE	reported	88xvfw	textfile	2	TRUE	NA	0.9741551	0.0258449	0.0001495	48.689084
10	15	78542897	0.009	3.61E-10	325625	exposure	TRUE	reported	88xvfw	textfile	2	TRUE	NA	0.9771372	0.0228628	0.0001311	42.684182
11	8	136533583	0.002	2.15E-08	326497	exposure	TRUE	reported	88xvfw	textfile	2	TRUE	NA	0.553678	0.446322	0.0001501	48.9997
12	21	39272184	0.004	1.09E-08	326497	exposure	TRUE	reported	88xvfw	textfile	2	TRUE	NA	0.874751	0.125249	8.60E-05	28.089828
13	15	56820815	0.003	6.52E-10	326497	exposure	TRUE	reported	88xvfw	textfile	2	TRUE	NA	0.218688	0.218688	0.0001215	39.689757
14	11	46498752	0.005	4.52E-11	326497	exposure	TRUE	reported	88xvfw	textfile	2	TRUE	NA	0.915507	0.084493	0.0001088	35.521382
15	20	32466899	0.003	1.69E-14	326497	exposure	TRUE	reported	88xvfw	textfile	2	TRUE	NA	0.622266	0.377734	0.0001254	40.959749
16	15	78828147	0.007	1.20E-13	325812	exposure	TRUE	reported	88xvfw	textfile	2	TRUE	NA	0.9761431	0.0238569	0.0001826	59.509839
17	16	89706498	0.003	2.04E-14	324557	exposure	TRUE	reported	88xvfw	textfile	2	FALSE	NA	0.428429	0.428429	0.0001275	41.387523
18	5	167147537	0.004	2.16E-08	326497	exposure	TRUE	reported	88xvfw	textfile	2	TRUE	NA	0.866799	0.133201	7.73E-05	25.25047
19	1	154576045	0.004	6.44E-13	324557	exposure	TRUE	reported	88xvfw	textfile	2	TRUE	NA	0.898608	0.101392	0.0001744	56.625276
20	1	77508845	0.003	2.42E-09	280416	exposure	TRUE	reported	88xvfw	textfile	2	TRUE	NA	0.549702	0.450298	0.000104	29.159792
21	7	32294030	0.003	2.81E-18	326497	exposure	TRUE	reported	88xvfw	textfile	2	TRUE	NA	0.332008	0.332008	0.0001723	56.249655
22	20	63355597	0.003	7.09E-24	320237	exposure	TRUE	reported	88xvfw	textfile	2	TRUE	NA	0.853877	0.146123	0.0004249	136.11026
23	3	136404933	0.003	1.78E-08	326497	exposure	TRUE	reported	88xvfw	textfile	2	TRUE	NA	0.269384	0.269384	8.82E-05	28.800935
24	9	133637247	0.003	6.01E-22	326497	exposure	TRUE	reported	88xvfw	textfile	2	TRUE	NA	0.819085	0.180915	0.0003206	104.72047
25	1	166890815	0.003	2.36E-08	324557	exposure	TRUE	reported	88xvfw	textfile	2	TRUE	NA	0.824056	0.175944	0.0001262	40.959748
26	19	40799625	0.007	1.21E-26	325689	exposure	TRUE	reported	88xvfw	textfile	2	TRUE	NA	0.9741551	0.0258449	0.0003969	129.30859
27	4	95187678	0.003	3.11E-08	326497	exposure	TRUE	reported	88xvfw	textfile	2	TRUE	NA	0.298211	0.298211	7.35E-05	24.009853
28	19	40847202	0.002	1.36E-137	326497	exposure	TRUE	reported	88xvfw	textfile	2	TRUE	NA	0.407555	0.407555	0.0029536	967.20408
29	20	11879643	0.003	1.22E-09	326497	exposure	TRUE	reported	88xvfw	textfile	2	TRUE	NA	0.579523	0.420477	7.97E-05	26.009841
30	1	18424989	0.002	1.55E-08	324557	exposure	TRUE	reported	88xvfw	textfile	2	FALSE	NA	0.486084	0.486084	0.000151	48.999698
31	16	69592233	0.003	1.69E-11	246711	exposure	TRUE	reported	88xvfw	textfile	2	TRUE	NA	0.576541	0.423459	0.0001695	41.817439
32	1	34919004	0.004	3.05E-08	322348	exposure	TRUE	reported	88xvfw	textfile	2	TRUE	NA	0.122266	0.122266	8.31E-05	26.780459
33	4	67009830	0.003	6.51E-10	300003	exposure	TRUE	reported	88xvfw	textfile	2	TRUE	NA	0.441352	0.441352	9.60E-05	28.800919
34	15	78427490	0.003	1.00E-200	326497	exposure	TRUE	reported	88xvfw	textfile	2	TRUE	NA	0.629225	0.370775	0.0025281	827.51604
35	19	4029786	0.003	1.46E-09	319050	exposure	TRUE	reported	88xvfw	textfile	2	TRUE	NA	0.815109	0.184891	0.0001492	47.609702
36	8	27584610	0.004	1.65E-14	326497	exposure	TRUE	reported	88xvfw	textfile	2	TRUE	NA	0.88668	0.11332	0.0001769	57.759646
37	2	60491212	0.002	8.42E-09	326497	exposure	TRUE	reported	88xvfw	textfile	2	TRUE	NA	0.582505	0.417495	0.0001588	51.839682
38	4	2995467	0.003	9.60E-11	281992	exposure	TRUE	reported	88xvfw	textfile	2	TRUE	NA	0.621272	0.378728	0.0001276	35.999745
39	7	74908852	0.004	1.82E-08	167512	exposure	TRUE	reported	88xvfw	textfile	2	TRUE	NA	0.253479	0.253479	0.0001855	31.080254
40	9	133574681	0.003	6.64E-10	300003	exposure	TRUE	reported	88xvfw	textfile	2	TRUE	NA	0.786282	0.213718	0.0001481	44.444148
41	12	109551053	0.003	7.93E-09	326497	exposure	TRUE	reported	88xvfw	textfile	2	TRUE	NA	0.823062	0.176938	0.0001215	39.689757
42	11	113578040	0.002	2.22E-12	326497	exposure	TRUE	reported	88xvfw	textfile	2	TRUE	NA	0.592445	0.407555	0.0002371	77.439526
43	11	16355573	0.003	5.23E-13	326497	exposure	TRUE	reported	88xvfw	textfile	2	TRUE	NA	0.680915	0.319085	0.0001281	41.817522
44	11	43673824	0.003	4.59E-10	326497	exposure	TRUE	reported	88xvfw	textfile	2	TRUE	NA	0.590457	0.409543	8.28E-05	27.039834
45	14	103691241	0.003	2.14E-11	326497	exposure	TRUE	reported	88xvfw	textfile	2	TRUE	NA	0.703777	0.296223	0.0001054	34.417567
46	6	26236840	0.003	1.24E-11	300003	exposure	TRUE	reported	88xvfw	textfile	2	TRUE	NA	0.704771	0.295229	0.0001394	41.817499
47	8	63706458	0.003	5.53E-12	326497	exposure	TRUE	reported	88xvfw	textfile	2	TRUE	NA	0.267396	0.267396	0.0001321	43.120847
48	13	111533936	0.002	6.50E-09	326497	exposure	TRUE	reported	88xvfw	textfile	2	FALSE	NA	0.480119	0.480119	0.0001588	51.839682
49	3	16805459	0.003	4.47E-09	326497	exposure	TRUE	reported	88xvfw	textfile	2	TRUE	NA	0.60835	0.39165	7.35E-05	24.009853

Smoking initiation

	...1	SNP	effect_allel	other_allel	effect_allel	other_allel	beta.exposu	beta.outcom	eaf.exposu	eaf.outcom	remove	palindromi	ambiguous	id.outcome	se.outcome	pval.outcom	ncase.outc	outcome	mr_keep.o	pval_origin	data_sourc	...22	...23	chr.exposu	pos.exposu	se.exposure	pval.exposu	ncase.expo	exposure	mr_keep.e	pval_origin	id.exposure	data_sourc	action	mr_keep	samplesize.	EAF2	MAF	PVE	FSTAT
1	1	rs10001365	A	G	A	G	-0.0152	-0.0654	0.378728	0.38	FALSE	FALSE	FALSE	9MV276	0.1582	0.6791	28243	outcome	TRUE	reported	textfile	174	18260	4	1.47E+08	0.002	4.32E-21	805431	exposure	TRUE	reported	iBwP7P	textfile	2	TRUE	NA	0.621272	0.378728	7.17E-05	57.75986
2	2	rs10062607	A	C	A	C	0.0103	0.0572	0.635189	0.63	FALSE	FALSE	FALSE	9MV276	0.1604	0.7214	28243	outcome	TRUE	reported	textfile	195	21282	5	79994811	0.002	2.28E-10	805431	exposure	TRUE	reported	iBwP7P	textfile	2	TRUE	NA	0.364811	0.364811	3.29E-05	26.52243
3	4	rs10233018	G	A	G	A	0.0139	-0.0119	0.512922	0.51	FALSE	FALSE	FALSE	9MV276	0.1564	0.9393	28243	outcome	TRUE	reported	textfile	217	24192	7	1.18E+08	0.002	1.49E-18	805431	exposure	TRUE	reported	iBwP7P	textfile	2	TRUE	NA	0.487078	0.487078	6.00E-05	48.30238
4	5	rs1025910	C	G	C	G	-0.0123	-0.1952	0.294235	0.29	FALSE	TRUE	FALSE	9MV276	0.1652	0.2373	28243	outcome	TRUE	reported	textfile	83	7504	18	52344970	0.002	4.83E-13	805431	exposure	TRUE	reported	iBwP7P	textfile	2	TRUE	NA	0.705765	0.294235	4.70E-05	37.82241
5	6	rs10279261	A	G	A	G	-0.0122	-0.2131	0.629225	0.63	FALSE	FALSE	FALSE	9MV276	0.1602	0.1835	28243	outcome	TRUE	reported	textfile	220	24780	7	1.34E+08	0.002	6.87E-14	805431	exposure	TRUE	reported	iBwP7P	textfile	2	TRUE	NA	0.370775	0.370775	4.62E-05	37.20991
6	7	rs10444314	G	T	G	T	-0.00922	-0.0609	0.428429	0.43	FALSE	FALSE	FALSE	9MV276	0.1578	0.6996	28243	outcome	TRUE	reported	textfile	27	3387	11	7929695	0.002	1.77E-08	780334	exposure	TRUE	reported	iBwP7P	textfile	2	TRUE	NA	0.571571	0.428429	2.72E-05	21.25205
7	8	rs10458563	G	A	G	A	0.0171	-0.2172	0.190855	0.19	FALSE	FALSE	FALSE	9MV276	0.1962	0.2682	28243	outcome	TRUE	reported	textfile	107	8769	1	50146578	0.002	2.17E-18	805431	exposure	TRUE	reported	iBwP7P	textfile	2	TRUE	NA	0.809145	0.190855	9.08E-05	73.10232
8	9	rs1050847	T	C	T	C	-0.0105	-0.0224	0.550696	0.55	FALSE	FALSE	FALSE	9MV276	0.1576	0.8868	28243	outcome	TRUE	reported	textfile	69	6512	16	87410128	0.002	3.71E-11	805431	exposure	TRUE	reported	iBwP7P	textfile	2	TRUE	NA	0.449304	0.449304	3.42E-05	27.56243
9	10	rs10698713	A	G	A	G	-0.0201	0.2549	0.052684	0.05	FALSE	FALSE	FALSE	9MV276	0.339	0.4521	28243	outcome	TRUE	reported	textfile	204	22518	6	1.58E+08	0.004	1.84E-08	805431	exposure	TRUE	reported	iBwP7P	textfile	2	TRUE	NA	0.947316	0.052684	3.13E-05	25.25056
10	11	rs10745324	G	A	G	A	-0.00962	-0.1489	0.672962	0.67	FALSE	FALSE	FALSE	9MV276	0.1627	0.3601	28243	outcome	TRUE	reported	textfile	92	7780	1	1.12E+08	0.002	1.97E-08	748334	exposure	TRUE	reported	iBwP7P	textfile	2	TRUE	NA	0.327038	0.327038	3.09E-05	23.13604
11	12	rs10753630	T	C	T	C	0.0109	0.0398	0.348907	0.35	FALSE	FALSE	FALSE	9MV276	0.1672	0.8121	28243	outcome	TRUE	reported	textfile	94	7868	1	1.64E+08	0.002	9.96E-11	780334	exposure	TRUE	reported	iBwP7P	textfile	2	TRUE	NA	0.651093	0.348907	3.81E-05	29.70242
12	13	rs10786721	A	C	A	C	0.0179	2.00E-04	0.416501	0.42	FALSE	FALSE	FALSE	9MV276	0.1562	0.9991	28243	outcome	TRUE	reported	textfile	2	716	10	1.03E+08	0.002	7.91E-27	748334	exposure	TRUE	reported	iBwP7P	textfile	2	TRUE	NA	0.583499	0.416501	0.000107	80.10229
13	14	rs1084445	C	T	C	T	-0.0121	0.0626	0.225646	0.22	FALSE	FALSE	FALSE	9MV276	0.1826	0.7317	28243	outcome	TRUE	reported	textfile	110	9213	1	72530838	0.002	1.80E-10	805431	exposure	TRUE	reported	iBwP7P	textfile	2	TRUE	NA	0.774354	0.225646	4.54E-05	36.60241
14	15	rs10927039	C	T	C	T	-0.0127	0.0978	0.196819	0.2	FALSE	FALSE	FALSE	9MV276	0.2046	0.6327	28243	outcome	TRUE	reported	textfile	103	8089	1	2.44E+08	0.002	2.39E-10	805431	exposure	TRUE	reported	iBwP7P	textfile	2	TRUE	NA	0.803181	0.196819	5.01E-05	40.3224
15	16	rs10950410	G	C	G	C	-0.0113	-0.0426	0.609344	0.6	FALSE	TRUE	FALSE	9MV276	0.1588	0.7885	28243	outcome	TRUE	reported	textfile	222	25032	7	1869450	0.002	1.54E-12	805431	exposure	TRUE	reported	iBwP7P	textfile	2	TRUE	NA	0.390656	0.390656	3.96E-05	31.92242
16	17	rs11012726	C	T	C	T	0.0143	-0.0133	0.33002	0.33	FALSE	FALSE	FALSE	9MV276	0.1675	0.9367	28243	outcome	TRUE	reported	textfile	9	1356	10	21508343	0.002	4.25E-17	805431	exposure	TRUE	reported	iBwP7P	textfile	2	TRUE	NA	0.66998	0.33002	6.35E-05	51.12237
17	18	rs11103667	T	C	T	C	0.0129	-0.129	0.178926	0.18	FALSE	FALSE	FALSE	9MV276	0.1975	0.5136	28243	outcome	TRUE	reported	textfile	242	27488	9	1.35E+08	0.002	1.47E-10	805431	exposure	TRUE	reported	iBwP7P	textfile	2	TRUE	NA	0.821074	0.178926	5.16E-05	41.6024
18	19	rs1111578	T	G	T	G	-0.0125	-0.214	0.15507	0.15	FALSE	FALSE	FALSE	9MV276	0.216	0.322	28243	outcome	TRUE	reported	textfile	29	3617	12	1.17E+08	0.002	8.22E-09	805431	exposure	TRUE	reported	iBwP7P	textfile	2	TRUE	NA	0.84493	0.15507	4.85E-05	39.0624
19	20	rs11130381	T	C	T	C	-0.00862	-0.0938	0.525845	0.53	FALSE	FALSE	FALSE	9MV276	0.1565	0.549	28243	outcome	TRUE	reported	textfile	163	16360	3	53815978	0.002	4.83E-08	805431	exposure	TRUE	reported	iBwP7P	textfile	2	TRUE	NA	0.474155	0.474155	2.31E-05	18.57605
20	21	rs11162019	T	C	T	C	-0.00971	-0.2605	0.379722	0.38	FALSE	FALSE	FALSE	9MV276	0.1606	0.1048	28243	outcome	TRUE	reported	textfile	115	10629	1	87447493	0.002	3.40E-09	805431	exposure	TRUE	reported	iBwP7P	textfile	2	TRUE	NA	0.620278	0.379722	2.93E-05	23.57097
21	22	rs11162976	C	A	C	A	0.0108	0.4936	0.306163	0.31	FALSE	FALSE	FALSE	9MV276	0.1751	0.004817	28243	outcome	TRUE	reported	textfile	113	10541	1	80356597	0.002	6.47E-10	805431	exposure	TRUE	reported	iBwP7P	textfile	2	TRUE	NA	0.693837	0.306163	3.62E-05	29.15993
22	23	rs11186174	A	G	A	G	-0.0179	0.4184	0.124254	0.12	FALSE	FALSE	FALSE	9MV276	0.2563	0.1025	28243	outcome	TRUE	reported	textfile	208	23373	6	50921306	0.003	3.16E-12	805431	exposure	TRUE	reported	iBwP7P	textfile	2	TRUE	NA	0.875746	0.124254	4.42E-05	35.60102
23	24	rs11192160	C	G	C	G	0.0139	-0.1384	0.185885	0.19	FALSE	TRUE	FALSE	9MV276	0.1982	0.485	28243	outcome	TRUE	reported	textfile	3	1159	10	1.05E+08	0.002	8.66E-10	630626	exposure	TRUE	reported	iBwP7P	textfile	2	TRUE	NA	0.814115	0.185885	7.66E-05	48.30235
24	25	rs11210229	G	A	G	A	-0.014	0.05	0.564612	0.56	FALSE	FALSE	FALSE	9MV276	0.1619	0.7574	28243	outcome	TRUE	reported	textfile	111	10183	1	73394345	0.002	4.94E-18	805431	exposure	TRUE	reported	iBwP7P	textfile	2	TRUE	NA	0.435388	0.435388	6.08E-05	48.99988
25	26	rs11258417	T	C	T	C	-0.0101	-0.1622	0.335984	0.34	FALSE	FALSE	FALSE	9MV276	0.16	0.3107	28243	outcome	TRUE	reported	textfile	8	1325	10	13491053	0.002	3.83E-10	805431	exposure	TRUE	reported	iBwP7P	textfile	2	TRUE	NA	0.664016	0.335984	3.17E-05	25.50244
26	27	rs1126757	T	C	T	C	0.0104	0.0294	0.472167	0.47	FALSE	FALSE	FALSE	9MV276	0.1578	0.852	28243	outcome	TRUE	reported	textfile	91	7777	19	55368504	0.002	4.15E-11	805431	exposure	TRUE	reported	iBwP7P	textfile	2	TRUE	NA	0.527833	0.472167	3.36E-05	27.03993
27	28	rs11405014	A	T	A	T	-0.0227	0.0659	0.06163	0.06	FALSE	TRUE	FALSE	9MV276	0.3327	0.8428	28243	outcome	TRUE	reported	textfile	156	15504	3	1.51E+08	0.003	2.56E-11	805431	exposure	TRUE	reported	iBwP7P	textfile	2	TRUE	NA	0.93837	0.06163	7.11E-05	57.2543
28	29	rs11490018	G	C	G	C	-0.0209	-0.226	0.058648	0.06	FALSE	TRUE	FALSE	9MV276	0.3038	0.4569	28243	outcome	TRUE	reported	textfile	67	6236	16	72595157	0.003	1.45E-12	805431	exposure	TRUE	reported	iBwP7P	textfile	2	TRUE	NA	0.941352	0.058648	6.03E-05	48.53432
29	30	rs1155641	A	G	A	G	0.0102	0.1037	0.333996	0.33	FALSE	FALSE	FALSE	9MV276	0.1649	0.5294	28243	outcome	TRUE	reported	textfile	56	5853	15	96959765	0.002	7.72E-10	805430	exposure	TRUE	reported	iBwP7P	textfile	2	TRUE	NA	0.666004	0.333996	3.23E-05	26.00994
30	31	rs11626595	T	C	T	C	-0.0151	0.284	0.156064	0.16	FALSE	FALSE	FALSE	9MV276	0.2117	0.1797	28243	outcome	TRUE	reported	textfile	44	4584	14	1.04E+08	0.002	2.73E-12	805431	exposure	TRUE	reported	iBwP7P	textfile	2	TRUE	NA	0.843936	0.156064	7.08E-05	57.00236
31	32	rs11632439	G	A	G	A	0.00898	0.0442	0.44831	0.45	FALSE	FALSE	FALSE	9MV276	0.1576	0.7793	28243	outcome	TRUE	reported	textfile	54	5614	15	80694671	0.002	1.31E-08	805430	exposure	TRUE	reported	iBwP7P	textfile	2	TRUE	NA	0.55169	0.44831	2.50E-05	20.16005
32	33	rs11656151	G	A	G	A	-0.0128	-0.2287	0.200795	0.2	FALSE	FALSE	FALSE	9MV276	0.1822	0.2094	28243	outcome	TRUE	reported	textfile	74	7015	17	45991126	0.002	5.61E-12	805431	exposure	TRUE	reported	iBwP7P	textfile	2	TRUE	NA	0.799205	0.200795	5.09E-05	40.9599
33	34	rs11673452	C	T	C	T	0.015	-0.101	0.11332	0.11	FALSE	FALSE	FALSE	9MV276	0.245	0.6801	28243	outcome	TRUE	reported	textfile	89	7743	19	33437095	0.003	3.40E-09	805431	exposure	TRUE	reported	iBwP7P	textfile	2	TRUE	NA	0.88668	0.11332	3.10E-05	24.99994
34	35	rs11693702	A	T	A	T	0.014	-0.1615	0.416501	0.42	FALSE	TRUE	FALSE	9MV276	0.1569	0.3034	28243	outcome	TRUE	reported	textfile	133	13205	2	1.62E+08	0.002	9.33E-19	805431	exposure	TRUE	reported	iBwP7P	textfile	2	TRUE	NA	0.583499	0.416501	6.08E-05	48.99988
35	36	rs11695197	A	G	A	G	0.0138	0.571	0.115308	0.11	FALSE	FALSE	FALSE	9MV276	0.2616	0.02907	28243	outcome	TRUE	reported	textfile	150	14817	2	97045684	0.002	6.28E-09	799280	exposure	TRUE	reported	iBwP7P	textfile	2	TRUE	NA	0.884692	0.115308	5.96E-05	47.60988
36	37	rs11716705	G	A	G	A	0.0117	-0.1227	0.267396	0.27	FALSE	FALSE	FALSE	9MV276	0.1783	0.4914	28243	outcome	TRUE	reported	textfile	160	15785	3	34687261	0.002	1.40E-10	805431	exposure	TRUE	reported	iBwP7P	textfile	2	TRUE	NA	0.732604	0.267396	4.25E-05	34.22242
37	38	rs11720703	T	C	T	C	0.0109	-0.4282	0.506958	0.51	FALSE	FALSE	FALSE	9MV276	0.1572	0.006439	28243	outcome	TRUE	reported	textfile	166	16409	3	71011489	0.002	5.58E-12	805431	exposure	TRUE	reported	iBwP7P	textfile	2	TRUE	NA	0.493042	0.493042	3.69E-05	29.70243
38	39	rs11742625	C	T	C	T	0.0157	0.0508	0.115308	0.11	FALSE	FALSE	FALSE	9MV276	0.2401	0.8323	28243	outcome	TRUE	reported	textfile	197	21609	5	92100296	0.002	2.19E-10	805431	exposure	TRUE	reported	iBwP7P	textfile	2	TRUE	NA	0.884692	0.115308	7.65E-05	61.62235
39	40	rs11756490	A	T	A	T	-0.0137	-0.0048	0.129225	0.13	FALSE	TRUE	FALSE	9MV276	0.2355	0.9836	28243	outcome	TRUE	reported	textfile	214	23948	6	99893898	0.002	6.20E-09	805431	exposure	TRUE	reported	iBwP7P	textfile	2	TRUE	NA	0.870775	0.129225	5.83E-05	46.92238
40	41	rs11865123	A	T	A	T	0.0106	-0.1513	0.27336	0.27	FALSE	TRUE	FALSE	9MV276	0.1761	0.3903	28243	outcome	TRUE	reported	textfile	64	5957	16	5790464	0.002	4.10E-08	697342	exposure	TRUE	reported	iBwP7P	textfile	2	TRUE	NA	0.72664	0.27336	4.03E-05	28.08992
41	42	rs11873164	T	C	T	C	-0.0155	-0.0208	0.131213	0.13	FALSE	FALSE	FALSE	9MV276	0.2166	0.9234	28243	outcome	TRUE	reported	textfile	82	7378	18	45079957	0.002	1.63E-12	805431	exposure	TRUE	reported	iBwP7P	textfile	2	TRUE	NA	0.868787	0.131213	7.46E-05	60.06235
42	43	rs11926232	G	A	G	A	0.0206	-0.2033	0.04672	0.05	FALSE	FALSE	FALSE	9MV276	0.3665	0.5791	28243	outcome	TRUE	reported	textfile	164	16362	3	55902389	0.004	1.16E-08	805431	exposure	TRUE	reported	iBwP7P	textfile	2	TRUE	NA	0.95328	0.04672	3.29E-05	26.52243
43	44	rs12027999	C	T	C	T	-0.0184	-0.4741	0.151093	0.15	FALSE	FALSE	FALSE	9MV276	0.2431	0.05111	28243	outcome	TRUE	reported	textfile	93	7825	1	1.54E+08	0.002	1.54E-13	732296	exposure	TRUE	reported	iBwP7P	textfile	2	TRUE	NA	0.848907	0.151093	0.000116	84.63977
44	45	rs12036050	C	T	C	T	0.0115	-0.1679	0.284294	0.28	FALSE	FALSE	FALSE	9MV276	0.1721	0.3293	28243	outcome	TRUE	reported	textfile	117	10833	1	96451935	0.002	7.18E-11	805431	exposure	TRUE	reported	iBwP7P	textfile	2	TRUE	NA	0.715706	0.284294	4.10E-05	33.06242
45	46	rs12079063	G	A	G	A	-0.00872	-0.0124	0.447316	0.45	FALSE	FALSE	FALSE	9MV276	0.1546	0.9361	28243	outcome	TRUE	reported	textfile	95	7935	1	1.74E+08	0.002	3.88E-08	805431	exposure	TRUE	reported	iBwP7P	textfile	2	TRUE	NA	0.552684	0.447316	2.36E-05	19.00955
46	47	rs12112638	G	A	G	A	-0.0122	0.1289	0.234592	0.23	FALSE	FALSE	FALSE	9MV276	0.1757	0.4632	28243	outcome	TRUE	reported	textfile	225	25793	7	70270265	0.002	8.74E-12	805431	exposure	TRUE	reported	iBwP7P	textfile	2	TRUE	NA	0.765408	0.234592	4.62E-05	37.20991
47	48	rs12133063	A	C	A	C	0.0116	-0.0427	0.371769	0.37	FALSE	FALSE	FALSE	9MV276	0.1631	0.7935	28243	outcome	TRUE	reported	textfile	116	10693	1	90749157	0.002	1.15E-12	805431	exposure	TRUE	reported	iBwP7P	textfile	2	TRUE	NA	0.628231	0.371769	4.18E-05	33.63992
48	50	rs12441907	A	C	A	C	-0.015	0.2803	0.166998	0.17	FALSE	FALSE	FALSE	9MV276	0.1957	0.1522	28243	outcome	TRUE	reported	textfile	55	5802	15	83253635	0.002	6.09E-14	805430	exposure	TRUE	reported	iBwP7P	textfile	2	TRUE	NA	0.833002	0.166998	6.98E-05	56.24986
49	51	rs1246265	C	T	C	T	0.0116	-0.2031	0.701789	0.7	FALSE	FALSE	FALSE	9MV276	0.169	0.2294	28243	outcome	TRUE	reported	textfile	248	27972	9	84146830	0.002	1.27E-11	805431	exposure	TRUE	reported	iBwP7P	textfile	2	TRUE	NA	0.298211	0.298211	4.18E-05	33.63992
50	52	rs12485391	A	C	A	C	0.0138	0.0213	0.12326	0.12	FALSE	FALSE	FALSE	9MV276	0.2406	0.9296	28243	outcome	TRUE	reported	textfile	159	15766	3	1.81E+08	0.002	1.89E-08	805431	exposure	TRUE	reported	iBwP7P	textfile	2	TRUE	NA	0.87674	0.12326	5.91E-05	47.60988
51	53	rs12600466	T	A	T	A	0.0115	-0.2135	0.301193	0.3	FALSE	TRUE	FALSE	9MV276	0.1673	0.2018	28243	outcome	TRUE	reported	textfile	75	7115	17	52229566	0.002	1.30E-11	805431	exposure	TRUE	reported	iBwP7P	textfile	2	TRUE	NA	0.698807	0.301193	4.10E-05	33.06242
52	54	rs12632110	G	A	G	A	-0.0138	0.125	0.663022	0.66	FALSE	FALSE	FALSE	9MV276	0.1644	0.4468	28243	outcome	TRUE	reported	textfile	162	16200	3	50186792	0.002	1.18E-16	805431	exposure	TRUE	reported	iBwP7P	textfile	2	TRUE	NA	0.336978	0.336978	5.91E-05	47.60988
53	55	rs12642744	T	G	T	G	-0.0124	-0.0882	0.757455	0.75	FALSE	FALSE	FALSE	9MV276	0.1813	0.6268	28243	outcome	TRUE	reported	textfile	177	18661	4	28025554	0.002	5.98E-11	748334	exposure	TRUE	reported	iBwP7P	textfile	2	TRUE	NA	0.242545	0.242545	5.14E-05	38.4399
54	56	rs12714017	C	T	C	T	0.0103	0.0635	0.532803	0.53	FALSE	FALSE	FALSE	9MV276	0.1555	0.6828	28243	outcome	TRUE	reported	textfile	149	14797	2	80772273	0.002	6.57E-10	723237	exposure	TRUE	reported	iBwP7P	textfile	2	TRUE	NA	0.467197	0.467197	3.67E-05	26.52243
55	57	rs12742446	T	G	T	G	0.0119	-0.1976	0.630219	0.63	FALSE	FALSE	FALSE	9MV276	0.1669	0.2365	28243	outcome	TRUE	reported	textfile	105	8258	1	33411456	0.002	2.74E-10	630626	exposure	TRUE	reported	iBwP7P	textfile	2	TRUE	NA	0.369781	0.369781	5.61E-05	35.40239
56	58	rs1291865	T	G	T	G	0.0116	-0.0412	0.531809	0.53	FALSE	FALSE	FALSE	9MV276	0.1546	0.79	28243	outcome	TRUE	reported	textfile	4	1167	10	11040229	0.002	2.12E-13	805431	exposure	TRUE	reported	iBwP7P	textfile	2	TRUE	NA	0.468191	0.468191	4.18E-05	33.63992
57	59	rs12923427	T	C	T	C	-0.0136	0.0309	0.219682	0.22	FALSE	FALSE	FALSE	9MV276	0.1867	0.8687	28243	outcome	TRUE	reported	textfile	59	5884	16	17481208	0.002	4.96E-12	805431	exposure	TRUE	reported	iBwP7P	textfile	2	TRUE	NA	0.780318	0.219682	5.74E-05	46.23989
58	60	rs12945403	C	T	C	T	-0.00941	-0.0633	0.616302	0.62	FALSE	FALSE	FALSE	9MV276	0.1655	0.7019	28243	outcome	TRUE	reported	textfile	78	7284	17	82046218	0.002	1.79E-08	804239	exposure	TRUE	reported	iBwP7P	textfile	2	TRUE	NA	0.383698	0.383698	2.75E-05	22.13697
59	61	rs13009008	G	A	G	A	-0.0097	-0.0475	0.651093	0.65	FALSE	FALSE	FALSE	9MV276	0.1641	0.772	28243	outcome	TRUE	reported	textfile	134	13260	2	1.73E+08	0.002	6.92E-09	805431	exposure	TRUE	reported	iBwP7P	textfile	2	TRUE	NA	0.348907	0.348907	2.92E-05	23.52244
60	62	rs13030994	A	G	A	G	0.0207	0.0565	0.508946	0.51	FALSE	FALSE	FALSE	9MV276	0.1567	0.7184	28243	outcome	TRUE	reported	textfile	131	12945	2	1.45E+08	0.002	2.42E-39	805431	exposure	TRUE	reported	iBwP7P	textfile	2	TRUE	NA	0.491054	0.491054	0.000133	107.1222
61	63	rs13145728	C	G	C	G	-0.0122	-0.0738	0.360835	0.36	FALSE	TRUE	FALSE	9MV276	0.1597	0.6441	28243	outcome	TRUE	reported	textfile	173	18107	4	1.4E+08	0.002	6.87E-14	805431	exposure	TRUE	reported	iBwP7P	textfile	2	TRUE	NA	0.639165	0.360835	4.62E-05	37.20991
62	64	rs13162305	T	A	T	A	0.00975	0.0176	0.33499	0.33	FALSE	TRUE	FALSE	9MV276	0.1639	0.9147	28243	outcome	TRUE	reported	textfile	185	19792	5	12122586	0.002	1.15E-08	780334	exposure	TRUE	reported	iBwP7P	textfile	2	TRUE	NA	0.66501	0.33499	3.05E-05	23.76556
63	66	rs13255625	G	A	G	A	0.0116	0.1989	0.695825	0.69	FALSE	FALSE	FALSE	9MV276	0.1642	0.2258	28243	outcome	TRUE	reported	textfile	232	26107	8	1.43E+08	0.002	4.03E-12	805431	exposure	TRUE	reported	iBwP7P	textfile	2	TRUE	NA	0.304175	0.304175	4.18E-05	33.63992
64	67	rs13261725	C	G	C	G	0.0131	-0.0116	0.261431	0.26	FALSE	TRUE	FALSE	9MV276	0.1784	0.948	28243	outcome	TRUE	reported	textfile	237	26512	8	90854069	0.002	3.43E-13	805431	exposure	TRUE	reported	iBwP7P	textfile	2	TRUE	NA	0.738569	0.261431	5.33E-05	42.90239
65	68	rs13301073	A	G	A	G	0.0109	-0.1048	0.348907	0.35	FALSE	FALSE	FALSE	9MV276	0.1597	0.5116	28243	outcome	TRUE	reported	textfile	241	27323	9	1.26E+08	0.002	3.13E-11	805431	exposure	TRUE	reported	iBwP7P	textfile	2	TRUE	NA	0.651093	0.348907	3.69E-05	29.70243
66	69	rs1334557	T	C	T	C	0.0132	0.005	0.245527	0.24	FALSE	FALSE	FALSE	9MV276	0.1808	0.9777	28243	outcome	TRUE	reported	textfile	14	1740	10	8748856	0.002	1.51E-12	805431	exposure	TRUE	reported	iBwP7P	textfile	2	TRUE	NA	0.754473	0.245527	5.41E-05	43.55989
67	71	rs13398418	A	T	A	T	0.0119	0.3864	0.259443	0.26	FALSE	TRUE	FALSE	9MV276	0.1798	0.03159	28243	outcome	TRUE	reported	textfile	137	13909	2	2.03E+08	0.002	2.09E-11	805431	exposure	TRUE	reported	iBwP7P	textfile	2	TRUE	NA	0.740557	0.259443	4.40E-05	35.40241
68	72	rs134529	C	T	C	T	-0.0116	0.1529	0.379722	0.38	FALSE	FALSE	FALSE	9MV276	0.1622	0.346	28243	outcome	TRUE	reported	textfile	125	11352	22	28385770	0.002	8.86E-13	805431	exposure	TRUE	reported	iBwP7P	textfile	2	TRUE	NA	0.620278	0.379722	4.18E-05	33.63992
69	73	rs1381287	T	C	T	C	0.0123	0.0505	0.50497	0.5	FALSE	FALSE	FALSE	9MV276	0.1558	0.7456	28243	outcome	TRUE	reported	textfile	49	4952	14	98131215	0.002	7.51E-15	805431	exposure	TRUE	reported	iBwP7P	textfile	2	TRUE	NA	0.49503	0.49503	4.70E-05	37.82241
70	74	rs1392446	T	C	T	C	0.00981	0.1481	0.532803	0.53	FALSE	FALSE	FALSE	9MV276	0.1554	0.3405	28243	outcome	TRUE	reported	textfile	191	20694	5	30831280	0.002	5.60E-10	805431	exposure	TRUE	reported	iBwP7P	textfile	2	TRUE	NA	0.467197	0.467197	2.99E-05	24.05897
71	75	rs1435679	A	G	A	G	0.01	-0.0606	0.581511	0.58	FALSE	FALSE	FALSE	9MV276	0.1618	0.7079	28243	outcome	TRUE	reported	textfile	50	5060	15	36107044	0.002	2.86E-10	805430	exposure	TRUE	reported	iBwP7P	textfile	2	TRUE	NA	0.418489	0.418489	3.10E-05	24.99994
72	76	rs14390987	C	T	C	T	0.0176	0.1302	0.107356	0.11	FALSE	FALSE	FALSE	9MV276	0.2468	0.5979	28243	outcome	TRUE	reported	textfile	145	14314	2	59067062	0.003	3.74E-12	805431	exposure	TRUE	reported	iBwP7P	textfile	2	TRUE	NA	0.892644	0.107356	4.27E-05	34.41769
73	77	rs14705217	T	G	T	G	0.0344	-0.7339	0.014911	0.01	FALSE	FALSE	FALSE	9MV276	0.586	0.2104	28243	outcome	TRUE	reported	textfile	96	7940	1	1.8E+08	0.006	1.31E-08	804537	exposure	TRUE	reported	iBwP7P	textfile	2	TRUE	NA	0.98509	0.014911	4.09E-05	32.87103
74	78	rs1485272	C	T	C	T	-0.00907	-0.2232	0.37674	0.38	FALSE	FALSE	FALSE	9MV276	0.1661	0.1791	28243	outcome	TRUE	reported	textfile	161	15787	3	3685905	0.002	4.07E-08	805431	exposure	TRUE	reported	iBwP7P	textfile	2	TRUE	NA	0.62326	0.37674	2.55E-05	20.56617
75	79	rs1490683	C	T	C	T	-0.00982	0.0347	0.333996	0.33	FALSE	FALSE	FALSE	9MV276	0.1604	0.8288	28243	outcome	TRUE	reported	textfile	175	18477	4	1.54E+08	0.002	2.84E-08	672245	exposure	TRUE	reported	iBwP7P	textfile	2	TRUE	NA	0.666004	0.333996	3.59E-05	24.10803
76	80	rs1499982	T	C	T	C	0.0216	-0.0028	0.835984	0.83	FALSE	FALSE	FALSE	9MV276	0.218	0.9897	28243	outcome	TRUE	reported	textfile	153	15225	3	1.18E+08	0.002	3.01E-23	805431	exposure	TRUE	reported	iBwP7P	textfile	2	TRUE	NA	0.164016	0.164016	0.000145	116.6397
77	81	rs1503211	A	G	A	G	0.00999	0.1459	0.454274	0.45	FALSE	FALSE	FALSE	9MV276	0.1575	0.3545	28243	outcome	TRUE	reported	textfile	182	19492	4	93158357	0.002	3.43E-10	805431	exposure	TRUE	reported	iBwP7P	textfile	2	TRUE	NA	0.545726	0.454274	3.10E-05	24.94996
78	82	rs1565735	A	T	A	T	-0.0232	-0.2324	0.199801	0.2	FALSE	TRUE	FALSE	9MV276	0.1916	0.2251	28243	outcome	TRUE	reported	textfile	233	26156	8	27568560	0.002	7.49E-32	805431	exposure	TRUE	reported	iBwP7P	textfile	2	TRUE	NA	0.800199	0.199801	0.000167	134.5597
79	83	rs160631	G	T	G	T	-0.0105	-0.0495	0.728628	0.72	FALSE	FALSE	FALSE	9MV276	0.1782	0.7813	28243	outcome	TRUE	reported	textfile	209	23406	6	53030432	0.002	3.56E-09	805431	exposure	TRUE	reported	iBwP7P	textfile	2	TRUE	NA	0.271372	0.271372	3.42E-05	27.56243
80	84	rs16896199	T	A	T	A	0.0118	-0.0286	0.166998	0.14	FALSE	TRUE	FALSE	9MV276	0.2013	0.8868	28243	outcome	TRUE	reported	textfile	210	23414	6	65078002	0.002	5.61E-09	805431	exposure	TRUE	reported	iBwP7P	textfile	2	TRUE	NA	0.833002	0.166998	4.32E-05	34.80991
81	85	rs16975171	A	C	A	C	-0.0172	-0.1689	0.10835	0.11	FALSE	FALSE	FALSE	9MV276	0.2773	0.5425	28243	outcome	TRUE	reported	textfile	81	7311	18	41689686	0.003	5.29E-09	805431	exposure	TRUE	reported	iBwP7P	textfile	2	TRUE	NA	0.89165	0.10835	4.08E-05	32.87103
82	86	rs172032	C	T	C	T	0.00901	0.1837	0.425447	0.42	FALSE	FALSE	FALSE	9MV276	0.1606	0.2525	28243	outcome	TRUE	reported	textfile	88	7739	19	18522945	0.002	1.56E-08	805431	exposure	TRUE	reported	iBwP7P	textfile	2	TRUE	NA	0.574553	0.425447	2.52E-05	20.29497
83	87	rs1733756	G	A	G	A	0.0101	0.0854	0.49503	0.49	FALSE	FALSE	FALSE	9MV276	0.1566	0.5856	28243	outcome	TRUE	reported	textfile	11	1571	10	54940461	0.002	1.23E-10	805431	exposure	TRUE	reported	iBwP7P	textfile	2	TRUE	NA	0.50497	0.49503	3.17E-05	25.50244
84	88	rs17432775	T	C	T	C	-0.0105	-0.1061	0.224652	0.22	FALSE	FALSE	FALSE	9MV276	0.1867	0.5697	28243	outcome	TRUE	reported	textfile	148	14648	2	63239328	0.002	2.55E-08	805431	exposure	TRUE	reported	iBwP7P	textfile	2	TRUE	NA	0.775348	0.224652	3.42E-05	27.56243
85	89	rs17594561	G	A	G	A	0.00966	-0.0034	0.416501	0.41	FALSE	FALSE	FALSE	9MV276	0.1555	0.9825	28243	outcome	TRUE	reported	textfile	48	4766	14	79152407	0.002	1.27E-09	805431	exposure	TRUE	reported	iBwP7P	textfile	2	TRUE	NA	0.583499	0.416501	2.90E-05	23.32884
86	90	rs1776631	C	T	C	T	-0.0106	0.3452	0.774354	0.77	FALSE	FALSE	FALSE	9MV276	0.1887	0.06728	28243	outcome	TRUE	reported	textfile	10	1551	10	31133648	0.002	3.92E-08	805431	exposure	TRUE	reported	iBwP7P	textfile	2	TRUE	NA	0.225646	0.225646	3.49E-05	28.08993
87	91	rs1899689	T	C	T	C	0.00909	0.0333	0.379722	0.38	FALSE	FALSE	FALSE	9MV276	0.1602	0.8355	28243	outcome	TRUE	reported	textfile	218	24314	7	1.22E+08	0.002	1.72E-08	805431	exposure	TRUE	reported	iBwP7P	textfile	2	TRUE	NA	0.620278	0.379722	2.56E-05	20.65697
88	93	rs1945737	C	T	C	T	-0.00968	0.0548	0.514911	0.51	FALSE	FALSE	FALSE	9MV276	0.1593	0.731	28243	outcome	TRUE	reported	textfile	84	7655	18	56077314	0.002	8.19E-10	805431	exposure	TRUE	reported	iBwP7P	textfile	2	TRUE	NA	0.485089	0.485089	2.91E-05	23.42554
89	94	rs1971318	T	C	T	C	0.0156	-0.1109	0.129225	0.13	FALSE	FALSE	FALSE	9MV276	0.2106	0.5984	28243	outcome	TRUE	reported	textfile	30	3696	12	1.21E+08	0.002	7.80E-13	805431	exposure	TRUE	reported	iBwP7P	textfile	2	TRUE	NA	0.870775	0.129225	7.55E-05	60.83985
90	95	rs1994247	T	G	T	G	0.0122	-0.0795	0.514911	0.51	FALSE	FALSE	FALSE	9MV276	0.1573	0.6133	28243	outcome	TRUE	reported	textfile	132	13118	2	1.55E+08	0.002	1.25E-14	805431	exposure	TRUE	reported	iBwP7P	textfile	2	TRUE	NA	0.485089	0.485089	4.62E-05	37.20991
91	96	rs2022815	A	G	A	G	0.0104	-0.2868	0.335984	0.34	FALSE	FALSE	FALSE	9MV276	0.1658	0.08364	28243	outcome	TRUE	reported	textfile	45	4617	14	27887527	0.002	1.12E-09	805431	exposure	TRUE	reported	iBwP7P	textfile	2	TRUE	NA	0.664016	0.335984	3.36E-05	27.03993
92	97	rs2072155	C	T	C	T	0.0128	0.2134	0.699801	0.7	FALSE	FALSE	FALSE	9MV276	0.1685	0.2054	28243	outcome	TRUE	reported	textfile	227	25920	7	78133140	0.002	1.16E-13	805431	exposure	TRUE	reported	iBwP7P	textfile	2	TRUE	NA	0.300199	0.300199	5.09E-05	40.9599

93	98	rs2135160	C	T	C	T	0.0157	-0.0451	0.091451	0.09	FALSE	FALSE	FALSE	9MV276	0.2612	0.8628	28243	outcome	TRUE	reported	textfile	138	13968	2	2.12E+08	0.003	8.23E-09	748334	exposure	TRUE	reported	iBwP7P	textfile	2	TRUE	NA	0.908549	0.091451	3.66E-05	27.3877
94	99	rs2163413	G	A	G	A	-0.0138	-0.0909	0.175944	0.18	FALSE	FALSE	FALSE	9MV276	0.2052	0.6577	28243	outcome	TRUE	reported	textfile	140	14043	2	2.25E+08	0.002	3.06E-11	796935	exposure	TRUE	reported	iBwP7P	textfile	2	TRUE	NA	0.824056	0.175944	5.97E-05	47.60988
95	100	rs2173019	A	T	A	T	0.0131	-0.1386	0.170974	0.17	FALSE	TRUE	FALSE	9MV276	0.2018	0.4922	28243	outcome	TRUE	reported	textfile	188	20163	5	1.68E+08	0.002	3.47E-10	805431	exposure	TRUE	reported	iBwP7P	textfile	2	TRUE	NA	0.829026	0.170974	5.33E-05	42.90239
96	101	rs2237303	A	G	A	G	-0.0102	0.3805	0.656064	0.66	FALSE	FALSE	FALSE	9MV276	0.1629	0.01953	28243	outcome	TRUE	reported	textfile	223	25083	7	21443987	0.002	6.37E-10	805431	exposure	TRUE	reported	iBwP7P	textfile	2	TRUE	NA	0.343936	0.343936	3.23E-05	26.00994
97	102	rs2289791	T	G	T	G	-0.0128	0.1979	0.240557	0.23	FALSE	FALSE	FALSE	9MV276	0.1791	0.2693	28243	outcome	TRUE	reported	textfile	53	5382	15	67184614	0.002	2.87E-12	805430	exposure	TRUE	reported	iBwP7P	textfile	2	TRUE	NA	0.759443	0.240557	5.09E-05	40.9599
98	103	rs2292239	G	T	G	T	0.0108	-0.0479	0.666998	0.66	FALSE	FALSE	FALSE	9MV276	0.1638	0.7697	28243	outcome	TRUE	reported	textfile	33	3792	12	56088396	0.002	1.03E-10	805431	exposure	TRUE	reported	iBwP7P	textfile	2	TRUE	NA	0.333002	0.333002	3.62E-05	29.15993
99	104	rs2313500	T	C	T	C	0.0128	0.2521	0.244533	0.25	FALSE	FALSE	FALSE	9MV276	0.2034	0.2152	28243	outcome	TRUE	reported	textfile	186	19820	5	1.55E+08	0.002	1.99E-12	805431	exposure	TRUE	reported	iBwP7P	textfile	2	TRUE	NA	0.755467	0.244533	5.09E-05	40.9599
100	105	rs2402821	A	G	A	G	0.0103	-0.036	0.62326	0.62	FALSE	FALSE	FALSE	9MV276	0.1566	0.818	28243	outcome	TRUE	reported	textfile	219	24596	7	1.27E+08	0.002	3.51E-09	697342	exposure	TRUE	reported	iBwP7P	textfile	2	TRUE	NA	0.37674	0.37674	3.80E-05	26.52242
101	106	rs2678903	G	A	G	A	0.0104	0.3282	0.591451	0.59	FALSE	FALSE	FALSE	9MV276	0.1583	0.03812	28243	outcome	TRUE	reported	textfile	144	14232	2	57910795	0.002	1.25E-10	805431	exposure	TRUE	reported	iBwP7P	textfile	2	TRUE	NA	0.408549	0.408549	3.36E-05	27.03993
102	107	rs2708630	T	C	T	C	-0.0102	-0.1452	0.691849	0.69	FALSE	FALSE	FALSE	9MV276	0.1666	0.3837	28243	outcome	TRUE	reported	textfile	114	10578	1	8387344	0.002	9.99E-10	805431	exposure	TRUE	reported	iBwP7P	textfile	2	TRUE	NA	0.308151	0.308151	3.23E-05	26.00994
103	108	rs2711607	T	G	T	G	0.0114	0.2028	0.157058	0.16	FALSE	FALSE	FALSE	9MV276	0.2071	0.3275	28243	outcome	TRUE	reported	textfile	52	5368	15	53825245	0.002	4.09E-08	805430	exposure	TRUE	reported	iBwP7P	textfile	2	TRUE	NA	0.842942	0.157058	4.03E-05	32.48992
104	109	rs2783130	G	A	G	A	-0.00877	-0.0351	0.502982	0.5	FALSE	FALSE	FALSE	9MV276	0.155	0.8207	28243	outcome	TRUE	reported	textfile	41	4543	13	79596025	0.002	2.71E-08	805431	exposure	TRUE	reported	iBwP7P	textfile	2	TRUE	NA	0.497018	0.497018	2.39E-05	19.22818
105	110	rs2876586	A	G	A	G	0.00905	0.2323	0.407555	0.41	FALSE	FALSE	FALSE	9MV276	0.1588	0.1435	28243	outcome	TRUE	reported	textfile	202	22490	6	1.45E+08	0.002	1.88E-08	805431	exposure	TRUE	reported	iBwP7P	textfile	2	TRUE	NA	0.592445	0.407555	2.54E-05	20.47557
106	111	rs288181	T	C	T	C	-0.00923	-0.0837	0.317097	0.31	FALSE	FALSE	FALSE	9MV276	0.1681	0.6183	28243	outcome	TRUE	reported	textfile	184	19759	5	1.08E+08	0.002	4.11E-08	805431	exposure	TRUE	reported	iBwP7P	textfile	2	TRUE	NA	0.682903	0.317097	2.64E-05	21.29817
107	112	rs2939756	A	G	A	G	-0.0103	-0.182	0.489066	0.49	FALSE	FALSE	FALSE	9MV276	0.1555	0.2419	28243	outcome	TRUE	reported	textfile	22	3310	11	41414747	0.002	6.87E-11	805431	exposure	TRUE	reported	iBwP7P	textfile	2	TRUE	NA	0.510934	0.489066	3.29E-05	26.52243
108	113	rs3110590	A	C	A	C	0.0111	-0.0967	0.248509	0.25	FALSE	FALSE	FALSE	9MV276	0.1777	0.5863	28243	outcome	TRUE	reported	textfile	80	7303	18	30228238	0.002	1.22E-09	805431	exposure	TRUE	reported	iBwP7P	textfile	2	TRUE	NA	0.751491	0.248509	3.82E-05	30.80242
109	114	rs3213876	C	T	C	T	0.0115	0.0381	0.315109	0.31	FALSE	FALSE	FALSE	9MV276	0.1635	0.8157	28243	outcome	TRUE	reported	textfile	85	7677	18	75472023	0.002	5.08E-12	805431	exposure	TRUE	reported	iBwP7P	textfile	2	TRUE	NA	0.684891	0.315109	4.10E-05	33.06242
110	115	rs326341	A	G	A	G	-0.0101	0.4109	0.462227	0.46	FALSE	FALSE	FALSE	9MV276	0.1558	0.008344	28243	outcome	TRUE	reported	textfile	151	14819	3	1.08E+08	0.002	6.16E-10	748334	exposure	TRUE	reported	iBwP7P	textfile	2	TRUE	NA	0.537773	0.462227	3.41E-05	25.50243
111	116	rs332827	A	G	A	G	-0.00893	-0.0913	0.452286	0.45	FALSE	FALSE	FALSE	9MV276	0.1656	0.5812	28243	outcome	TRUE	reported	textfile	108	8797	1	61277488	0.002	2.81E-08	780334	exposure	TRUE	reported	iBwP7P	textfile	2	TRUE	NA	0.547714	0.452286	2.55E-05	19.93617
112	117	rs34367058	T	C	T	C	0.0138	-0.2587	0.170974	0.17	FALSE	FALSE	FALSE	9MV276	0.1891	0.1713	28243	outcome	TRUE	reported	textfile	130	12483	2	1.37E+08	0.002	1.36E-13	805431	exposure	TRUE	reported	iBwP7P	textfile	2	TRUE	NA	0.829026	0.170974	5.91E-05	47.60988
113	118	rs34488670	C	T	C	T	0.0176	-0.1623	0.206759	0.21	FALSE	FALSE	FALSE	9MV276	0.1893	0.3912	28243	outcome	TRUE	reported	textfile	51	5260	15	47392739	0.002	8.73E-20	805430	exposure	TRUE	reported	iBwP7P	textfile	2	TRUE	NA	0.793241	0.206759	9.61E-05	77.43981
114	119	rs35891966	A	G	A	G	-0.019	-0.1058	0.071571	0.07	FALSE	FALSE	FALSE	9MV276	0.3067	0.7301	28243	outcome	TRUE	reported	textfile	20	2960	11	20107765	0.003	6.52E-10	805431	exposure	TRUE	reported	iBwP7P	textfile	2	TRUE	NA	0.928429	0.071571	4.98E-05	40.11101
115	120	rs3781295	A	G	A	G	-0.0116	0.2714	0.387674	0.38	FALSE	FALSE	FALSE	9MV276	0.1611	0.09208	28243	outcome	TRUE	reported	textfile	1	206	10	1.02E+08	0.002	1.13E-12	805431	exposure	TRUE	reported	iBwP7P	textfile	2	TRUE	NA	0.612326	0.387674	4.18E-05	33.63992
116	121	rs3801289	C	A	C	A	-0.0115	0.1933	0.363817	0.36	FALSE	FALSE	FALSE	9MV276	0.1646	0.2402	28243	outcome	TRUE	reported	textfile	229	25951	7	97008955	0.002	3.64E-12	805431	exposure	TRUE	reported	iBwP7P	textfile	2	TRUE	NA	0.636183	0.363817	4.10E-05	33.06242
117	122	rs3814994	T	G	T	G	0.00962	0.0434	0.373757	0.37	FALSE	FALSE	FALSE	9MV276	0.1621	0.789	28243	outcome	TRUE	reported	textfile	228	25935	7	88798071	0.002	7.44E-09	805431	exposure	TRUE	reported	iBwP7P	textfile	2	TRUE	NA	0.626243	0.373757	2.87E-05	23.13604
118	123	rs3895907	G	A	G	A	-0.0129	-0.1279	0.548708	0.55	FALSE	FALSE	FALSE	9MV276	0.156	0.4124	28243	outcome	TRUE	reported	textfile	112	10424	1	74540343	0.002	1.56E-14	723237	exposure	TRUE	reported	iBwP7P	textfile	2	TRUE	NA	0.451292	0.451292	5.75E-05	41.60238
119	124	rs3905125	T	C	T	C	0.0104	0.2819	0.542744	0.54	FALSE	FALSE	FALSE	9MV276	0.1584	0.07516	28243	outcome	TRUE	reported	textfile	101	8049	1	2.37E+08	0.002	4.28E-11	805431	exposure	TRUE	reported	iBwP7P	textfile	2	TRUE	NA	0.457256	0.457256	3.36E-05	27.03993
120	125	rs3934797	A	G	A	G	-0.0151	0.1048	0.171968	0.17	FALSE	FALSE	FALSE	9MV276	0.2063	0.6114	28243	outcome	TRUE	reported	textfile	172	18012	4	1.12E+08	0.002	1.90E-13	805431	exposure	TRUE	reported	iBwP7P	textfile	2	TRUE	NA	0.828032	0.171968	7.08E-05	57.00236
121	126	rs39784	A	C	A	C	0.0115	0.3864	0.722664	0.72	FALSE	FALSE	FALSE	9MV276	0.1761	0.0282	28243	outcome	TRUE	reported	textfile	189	20660	5	1.71E+08	0.002	6.41E-11	805431	exposure	TRUE	reported	iBwP7P	textfile	2	TRUE	NA	0.277336	0.277336	4.10E-05	33.06242
122	127	rs404263	T	C	T	C	-0.0112	0.1716	0.65507	0.65	FALSE	FALSE	FALSE	9MV276	0.1617	0.2885	28243	outcome	TRUE	reported	textfile	31	3720	12	1.25E+08	0.002	1.55E-10	697342	exposure	TRUE	reported	iBwP7P	textfile	2	TRUE	NA	0.34493	0.34493	4.50E-05	31.35991
123	128	rs4044321	G	A	G	A	-0.0164	-0.2424	0.668986	0.66	FALSE	FALSE	FALSE	9MV276	0.164	0.1393	28243	outcome	TRUE	reported	textfile	187	20073	5	1.68E+08	0.002	3.04E-23	805431	exposure	TRUE	reported	iBwP7P	textfile	2	TRUE	NA	0.331014	0.331014	8.35E-05	67.23983
124	129	rs4374330	T	C	T	C	0.0127	0.1765	0.764414	0.76	FALSE	FALSE	FALSE	9MV276	0.1814	0.3305	28243	outcome	TRUE	reported	textfile	135	13576	2	1.81E+08	0.002	3.73E-12	805431	exposure	TRUE	reported	iBwP7P	textfile	2	TRUE	NA	0.235586	0.235586	5.01E-05	40.3224
125	131	rs4479577	T	C	T	C	0.00898	-0.0946	0.498012	0.5	FALSE	FALSE	FALSE	9MV276	0.1567	0.5462	28243	outcome	TRUE	reported	textfile	165	16369	3	5682131	0.002	2.11E-08	780334	exposure	TRUE	reported	iBwP7P	textfile	2	TRUE	NA	0.501988	0.498012	2.58E-05	20.16005
126	132	rs4543592	C	T	C	T	0.012	-0.006	0.49006	0.48	FALSE	FALSE	FALSE	9MV276	0.1571	0.9696	28243	outcome	TRUE	reported	textfile	245	27525	9	3014254	0.002	2.51E-14	805431	exposure	TRUE	reported	iBwP7P	textfile	2	TRUE	NA	0.50994	0.49006	4.47E-05	35.99991
127	133	rs4571506	T	C	T	C	-0.0151	-0.0303	0.481113	0.48	FALSE	FALSE	FALSE	9MV276	0.153	0.8432	28243	outcome	TRUE	reported	textfile	196	21508	5	88461101	0.002	1.64E-21	805431	exposure	TRUE	reported	iBwP7P	textfile	2	TRUE	NA	0.518887	0.481113	7.08E-05	57.00236
128	134	rs4579569	A	G	A	G	0.0122	0.0443	0.499006	0.5	FALSE	FALSE	FALSE	9MV276	0.1551	0.7751	28243	outcome	TRUE	reported	textfile	235	26303	8	64001205	0.002	1.23E-14	805431	exposure	TRUE	reported	iBwP7P	textfile	2	TRUE	NA	0.500994	0.499006	4.62E-05	37.20991
129	135	rs465646	A	G	A	G	-0.0236	0.1742	0.849901	0.85	FALSE	FALSE	FALSE	9MV276	0.209	0.4047	28243	outcome	TRUE	reported	textfile	200	22253	6	1.11E+08	0.002	1.70E-28	805431	exposure	TRUE	reported	iBwP7P	textfile	2	TRUE	NA	0.150099	0.150099	0.000173	139.2397
130	136	rs4659805	T	G	T	G	0.00936	-0.0411	0.605368	0.52	FALSE	FALSE	FALSE	9MV276	0.161	0.7986	28243	outcome	TRUE	reported	textfile	102	8077	1	2.38E+08	0.002	8.36E-09	805431	exposure	TRUE	reported	iBwP7P	textfile	2	TRUE	NA	0.394632	0.394632	2.72E-05	21.90235
131	137	rs4751614	T	A	T	A	0.0122	0.0309	0.23161	0.23	FALSE	TRUE	FALSE	9MV276	0.1826	0.8658	28243	outcome	TRUE	reported	textfile	5	1240	10	1.17E+08	0.002	6.35E-11	805431	exposure	TRUE	reported	iBwP7P	textfile	2	TRUE	NA	0.76839	0.23161	4.62E-05	37.20991
132	138	rs4785187	A	G	A	G	0.0119	-0.0312	0.244533	0.24	FALSE	FALSE	FALSE	9MV276	0.1884	0.8687	28243	outcome	TRUE	reported	textfile	63	5946	16	49732861	0.002	2.03E-10	805431	exposure	TRUE	reported	iBwP7P	textfile	2	TRUE	NA	0.755467	0.244533	4.40E-05	35.40241
133	139	rs4819027	G	C	G	C	-0.00989	0.14	0.718688	0.72	FALSE	TRUE	FALSE	9MV276	0.172	0.4157	28243	outcome	TRUE	reported	textfile	123	11348	21	45075309	0.002	1.34E-08	805431	exposure	TRUE	reported	iBwP7P	textfile	2	TRUE	NA	0.281312	0.281312	3.04E-05	24.45296
134	140	rs4857114	T	C	T	C	0.00881	-0.1725	0.406561	0.4	FALSE	FALSE	FALSE	9MV276	0.1581	0.2754	28243	outcome	TRUE	reported	textfile	171	17895	3	94494239	0.002	3.03E-08	805431	exposure	TRUE	reported	iBwP7P	textfile	2	TRUE	NA	0.593439	0.406561	2.41E-05	19.40398
135	141	rs4888444	G	A	G	A	-0.0245	0.155	0.039761	0.04	FALSE	FALSE	FALSE	9MV276	0.3732	0.678	28243	outcome	TRUE	reported	textfile	68	6312	16	75656381	0.004	6.84E-10	805431	exposure	TRUE	reported	iBwP7P	textfile	2	TRUE	NA	0.960239	0.039761	4.66E-05	37.51553
136	142	rs4944844	C	T	C	T	-0.0133	-0.241	0.84493	0.83	FALSE	FALSE	FALSE	9MV276	0.2095	0.25	28243	outcome	TRUE	reported	textfile	26	3384	11	73600660	0.002	2.33E-10	805431	exposure	TRUE	reported	iBwP7P	textfile	2	TRUE	NA	0.15507	0.15507	5.49E-05	44.22239
137	143	rs540356	A	C	A	C	0.0116	0.1216	0.370775	0.37	FALSE	FALSE	FALSE	9MV276	0.1579	0.4413	28243	outcome	TRUE	reported	textfile	19	2915	11	1.32E+08	0.002	4.07E-13	805431	exposure	TRUE	reported	iBwP7P	textfile	2	TRUE	NA	0.629225	0.370775	4.18E-05	33.63992
138	144	rs540860	G	A	G	A	0.0117	0.0491	0.562624	0.55	FALSE	FALSE	FALSE	9MV276	0.1568	0.7541	28243	outcome	TRUE	reported	textfile	17	2634	11	1.22E+08	0.002	3.18E-13	780334	exposure	TRUE	reported	iBwP7P	textfile	2	TRUE	NA	0.437376	0.437376	4.39E-05	34.22241
139	145	rs551739	T	A	T	A	0.0102	0.2479	0.694831	0.69	FALSE	TRUE	FALSE	9MV276	0.169	0.1423	28243	outcome	TRUE	reported	textfile	32	3749	12	40020273	0.002	4.42E-09	805431	exposure	TRUE	reported	iBwP7P	textfile	2	TRUE	NA	0.305169	0.305169	3.23E-05	26.00994
140	146	rs557544	C	T	C	T	-0.00945	0.0734	0.356859	0.35	FALSE	FALSE	FALSE	9MV276	0.1698	0.6654	28243	outcome	TRUE	reported	textfile	199	22209	6	1.09E+08	0.002	2.82E-08	805431	exposure	TRUE	reported	iBwP7P	textfile	2	TRUE	NA	0.643141	0.356859	2.77E-05	22.32557
141	147	rs55921136	C	T	C	T	-0.0136	-0.2854	0.240557	0.24	FALSE	FALSE	FALSE	9MV276	0.1917	0.1366	28243	outcome	TRUE	reported	textfile	99	7967	1	2.1E+08	0.002	1.90E-12	805431	exposure	TRUE	reported	iBwP7P	textfile	2	TRUE	NA	0.759443	0.240557	5.74E-05	46.23989
142	148	rs56169608	A	G	A	G	0.00906	0.092	0.441352	0.44	FALSE	FALSE	FALSE	9MV276	0.155	0.5529	28243	outcome	TRUE	reported	textfile	97	7944	1	18113497	0.002	1.00E-08	805431	exposure	TRUE	reported	iBwP7P	textfile	2	TRUE	NA	0.558648	0.441352	2.55E-05	20.52085
143	149	rs56225373	G	T	G	T	0.016	-0.0237	0.158052	0.16	FALSE	FALSE	FALSE	9MV276	0.2209	0.9147	28243	outcome	TRUE	reported	textfile	58	5871	16	13661551	0.003	3.13E-08	500394	exposure	TRUE	reported	iBwP7P	textfile	2	TRUE	NA	0.841948	0.158052	5.68E-05	28.44433
144	150	rs56348592	G	A	G	A	0.0126	0.3573	0.142147	0.14	FALSE	FALSE	FALSE	9MV276	0.2199	0.1043	28243	outcome	TRUE	reported	textfile	243	27495	9	16749267	0.002	1.81E-08	799280	exposure	TRUE	reported	iBwP7P	textfile	2	TRUE	NA	0.857853	0.142147	4.97E-05	39.6899
145	151	rs56820925	T	C	T	C	-0.0108	0.077	0.354871	0.35	FALSE	FALSE	FALSE	9MV276	0.1614	0.6335	28243	outcome	TRUE	reported	textfile	121	11272	20	55812318	0.002	2.25E-10	744041	exposure	TRUE	reported	iBwP7P	textfile	2	TRUE	NA	0.645129	0.354871	3.92E-05	29.15992
146	152	rs574835	A	G	A	G	-0.0103	0.0267	0.365805	0.36	FALSE	FALSE	FALSE	9MV276	0.161	0.8682	28243	outcome	TRUE	reported	textfile	24	3340	11	64343196	0.002	2.33E-10	805431	exposure	TRUE	reported	iBwP7P	textfile	2	TRUE	NA	0.634195	0.365805	3.29E-05	26.52243
147	153	rs5751239	T	C	T	C	-0.0102	-0.0168	0.527833	0.53	FALSE	FALSE	FALSE	9MV276	0.156	0.9145	28243	outcome	TRUE	reported	textfile	126	11528	22	42196233	0.002	1.02E-10	805431	exposure	TRUE	reported	iBwP7P	textfile	2	TRUE	NA	0.472167	0.472167	3.23E-05	26.00994
148	154	rs58400863	A	G	A	G	-0.0114	-0.1273	0.385686	0.38	FALSE	FALSE	FALSE	9MV276	0.1637	0.4368	28243	outcome	TRUE	reported	textfile	178	19131	4	31182862	0.002	5.03E-12	805431	exposure	TRUE	reported	iBwP7P	textfile	2	TRUE	NA	0.614314	0.385686	4.03E-05	32.48992
149	155	rs58547189	A	G	A	G	-0.0106	0.144	0.245527	0.24	FALSE	FALSE	FALSE	9MV276	0.1808	0.4257	28243	outcome	TRUE	reported	textfile	87	7731	19	18351898	0.002	1.73E-08	732296	exposure	TRUE	reported	iBwP7P	textfile	2	TRUE	NA	0.754473	0.245527	3.84E-05	28.08992
150	156	rs60453921	A	T	A	T	0.0127	-0.071	0.150099	0.15	FALSE	TRUE	FALSE	9MV276	0.2067	0.7314	28243	outcome	TRUE	reported	textfile	13	1711	10	85602822	0.002	1.06E-09	805431	exposure	TRUE	reported	iBwP7P	textfile	2	TRUE	NA	0.849901	0.150099	5.01E-05	40.3224
151	157	rs6088618	A	G	A	G	-0.0108	-0.0121	0.4334	0.43	FALSE	FALSE	FALSE	9MV276	0.1572	0.9388	28243	outcome	TRUE	reported	textfile	120	11250	20	34821547	0.002	5.89E-11	744041	exposure	TRUE	reported	iBwP7P	textfile	2	TRUE	NA	0.5666	0.4334	3.92E-05	29.15992
152	158	rs6141314	A	G	A	G	0.0131	-0.1892	0.215706	0.21	FALSE	FALSE	FALSE	9MV276	0.1817	0.2977	28243	outcome	TRUE	reported	textfile	119	10916	20	32505711	0.002	3.59E-13	801138	exposure	TRUE	reported	iBwP7P	textfile	2	TRUE	NA	0.784294	0.215706	5.35E-05	42.90239
153	159	rs61533748	C	T	C	T	0.0104	-0.2456	0.377734	0.38	FALSE	FALSE	FALSE	9MV276	0.1618	0.1291	28243	outcome	TRUE	reported	textfile	139	13983	2	22360096	0.002	1.50E-10	805431	exposure	TRUE	reported	iBwP7P	textfile	2	TRUE	NA	0.622266	0.377734	3.36E-05	27.03993
154	160	rs61959481	A	G	A	G	-0.0123	0.2315	0.203777	0.2	FALSE	FALSE	FALSE	9MV276	0.195	0.2352	28243	outcome	TRUE	reported	textfile	37	3870	13	55260794	0.002	2.06E-10	805431	exposure	TRUE	reported	iBwP7P	textfile	2	TRUE	NA	0.796223	0.203777	4.70E-05	37.82241
155	161	rs62107261	C	T	C	T	-0.0259	-0.0736	0.044732	0.05	FALSE	FALSE	FALSE	9MV276	0.364	0.8398	28243	outcome	TRUE	reported	textfile	141	14053	2	422144	0.004	2.13E-11	805431	exposure	TRUE	reported	iBwP7P	textfile	2	TRUE	NA	0.955268	0.044732	5.21E-05	41.92552
156	162	rs62135525	T	C	T	C	-0.0221	0.6332	0.038767	0.04	FALSE	FALSE	FALSE	9MV276	0.3699	0.08696	28243	outcome	TRUE	reported	textfile	142	14067	2	44072740	0.004	4.61E-09	805431	exposure	TRUE	reported	iBwP7P	textfile	2	TRUE	NA	0.961233	0.038767	3.79E-05	30.52555
157	163	rs62181771	A	G	A	G	0.00989	-0.0198	0.559642	0.56	FALSE	FALSE	FALSE	9MV276	0.1578	0.9002	28243	outcome	TRUE	reported	textfile	136	13737	2	2E+08	0.002	4.47E-10	805431	exposure	TRUE	reported	iBwP7P	textfile	2	TRUE	NA	0.440358	0.440358	3.04E-05	24.45296
158	164	rs62250713	G	A	G	A	-0.0189	0.0622	0.633201	0.63	FALSE	FALSE	FALSE	9MV276	0.162	0.7012	28243	outcome	TRUE	reported	textfile	169	16838	3	85464643	0.002	1.63E-30	805431	exposure	TRUE	reported	iBwP7P	textfile	2	TRUE	NA	0.366799	0.366799	0.000111	89.30228
159	165	rs62254171	A	G	A	G	0.031	0.0946	0.014911	0.01	FALSE	FALSE	FALSE	9MV276	0.4977	0.8493	28243	outcome	TRUE	reported	textfile	168	16524	3	84575615	0.005	3.73E-09	800208	exposure	TRUE	reported	iBwP7P	textfile	2	TRUE	NA	0.98509	0.014911	4.80E-05	38.4399
160	166	rs62258903	G	C	G	C	-0.0158	0.0946	0.127237	0.13	FALSE	TRUE	FALSE	9MV276	0.2627	0.7187	28243	outcome	TRUE	reported	textfile	167	16457	3	75053282	0.002	7.20E-12	805431	exposure	TRUE	reported	iBwP7P	textfile	2	TRUE	NA	0.872763	0.127237	7.75E-05	62.40985
161	167	rs62266876	G	C	G	C	0.0154	-0.4434	0.072565	0.07	FALSE	TRUE	FALSE	9MV276	0.2706	0.1012	28243	outcome	TRUE	reported	textfile	154	15362	3	1.29E+08	0.003	3.06E-08	805431	exposure	TRUE	reported	iBwP7P	textfile	2	TRUE	NA	0.927435	0.072565	3.27E-05	26.35105
162	168	rs62419578	A	T	A	T	0.0131	-0.1039	0.122266	0.12	FALSE	TRUE	FALSE	9MV276	0.2323	0.6548	28243	outcome	TRUE	reported	textfile	212	23694	6	83647240	0.002	1.08E-08	805431	exposure	TRUE	reported	iBwP7P	textfile	2	TRUE	NA	0.877734	0.122266	5.33E-05	42.90239
163	169	rs6265	T	C	T	C	-0.0199	0.0509	0.196819	0.19	FALSE	FALSE	FALSE	9MV276	0.1967	0.7959	28243	outcome	TRUE	reported	textfile	21	3077	11	27658369	0.002	1.86E-22	805431	exposure	TRUE	reported	iBwP7P	textfile	2	TRUE	NA	0.803181	0.196819	0.000123	99.00225
164	170	rs6438208	A	G	A	G	-0.0104	-0.0354	0.248509	0.25	FALSE	FALSE	FALSE	9MV276	0.1781	0.8425	28243	outcome	TRUE	reported	textfile	152	14885	3	1.14E+08	0.002	7.33E-09	805431	exposure	TRUE	reported	iBwP7P	textfile	2	TRUE	NA	0.751491	0.248509	3.36E-05	27.03993
165	171	rs6464024	T	C	T	C	-0.0121	0.0519	0.437376	0.44	FALSE	FALSE	FALSE	9MV276	0.1583	0.7431	28243	outcome	TRUE	reported	textfile	221	24865	7	1648733	0.002	3.38E-14	805431	exposure	TRUE	reported	iBwP7P	textfile	2	TRUE	NA	0.562624	0.437376	4.54E-05	36.60241
166	172	rs6472232	G	T	G	T	-0.00942	0.0169	0.375746	0.38	FALSE	FALSE	FALSE	9MV276	0.1635	0.9176	28243	outcome	TRUE	reported	textfile	236	26458	8	65880397	0.002	1.43E-08	805431	exposure	TRUE	reported	iBwP7P	textfile	2	TRUE	NA	0.624254	0.375746	2.75E-05	22.18404
167	173	rs6497840	A	G	A	G	0.0128	0.2322	0.713718	0.71	FALSE	FALSE	FALSE	9MV276	0.1746	0.1835	28243	outcome	TRUE	reported	textfile	61	5915	16	25340312	0.002	1.13E-12	748334	exposure	TRUE	reported	iBwP7P	textfile	2	TRUE	NA	0.286282	0.286282	5.47E-05	40.95989
168	174	rs6598539	C	T	C	T	0.011	0.0306	0.512922	0.51	FALSE	FALSE	FALSE	9MV276	0.1561	0.8448	28243	outcome	TRUE	reported	textfile	57	5869	15	98661254	0.002	5.76E-12	780333	exposure	TRUE	reported	iBwP7P	textfile	2	TRUE	NA	0.487078	0.487078	3.88E-05	30.24992
169	176	rs66680800	T	G	T	G	-0.0125	0.0566	0.430418	0.43	FALSE	FALSE	FALSE	9MV276	0.1581	0.7205	28243	outcome	TRUE	reported	textfile	170	17671	3	85936174	0.002	1.13E-14	805431	exposure	TRUE	reported	iBwP7P	textfile	2	TRUE	NA	0.569582	0.430418	4.85E-05	39.0624
170	177	rs6705147	T	C	T	C	0.00986	-0.0307	0.322068	0.31	FALSE	FALSE	FALSE	9MV276	0.1745	0.8603	28243	outcome	TRUE	reported	textfile	129	12279	2	1.32E+08	0.002	5.24E-09	805431	exposure	TRUE	reported	iBwP7P	textfile	2	TRUE	NA	0.677932	0.322068	3.02E-05	24.30484
171	178	rs6728726	C	T	C	T	0.0187	0.1215	0.83002	0.83	FALSE	FALSE	FALSE	9MV276	0.2067	0.5567	28243	outcome	TRUE	reported	textfile	147	14573	2	623976	0.002	4.27E-19	805431	exposure	TRUE	reported	iBwP7P	textfile	2	TRUE	NA	0.16998	0.16998	0.000109	87.42228
172	179	rs6852117	G	C	G	C	-0.0118	0.1364	0.40159	0.4	FALSE	TRUE	FALSE	9MV276	0.159	0.3909	28243	outcome	TRUE	reported	textfile	176	18567	4	1.72E+08	0.002	1.97E-13	805431	exposure	TRUE	reported	iBwP7P	textfile	2	TRUE	NA	0.59841	0.40159	4.32E-05	34.80991
173	180	rs6861333	G	C	G	C	-0.0106	0.128	0.357853	0.36	FALSE	TRUE	FALSE	9MV276	0.1588	0.4203	28243	outcome	TRUE	reported	textfile	193	20738	5	51408099	0.002	3.12E-10	748334	exposure	TRUE	reported	iBwP7P	textfile	2	TRUE	NA	0.642147	0.357853	3.75E-05	28.08992
174	181	rs6868892	T	C	T	C	0.0101	-0.0412	0.50497	0.5	FALSE	FALSE	FALSE	9MV276	0.1569	0.7929	28243	outcome	TRUE	reported	textfile	190	20686	5	22193671	0.002	1.92E-08	624207	exposure	TRUE	reported	iBwP7P	textfile	2	TRUE	NA	0.49503	0.49503	4.09E-05	25.50242
175	182	rs6959670	T	C	T	C	0.00987	0.2045	0.355865	0.35	FALSE	FALSE	FALSE	9MV276	0.1672	0.2213	28243	outcome	TRUE	reported	textfile	215	23996	7	1.11E+08	0.002	7.29E-09	805431	exposure	TRUE	reported	iBwP7P	textfile	2	TRUE	NA	0.644135	0.355865	3.02E-05	24.35416
176	183	rs6973168	G	C	G	C	-0.00946	-0.0969	0.590457	0.59	FALSE	TRUE	FALSE	9MV276	0.1577	0.5387	28243	outcome	TRUE	reported	textfile	216	24098	7	1.15E+08	0.002	2.70E-09	805431	exposure	TRUE	reported	iBwP7P	textfile	2	TRUE	NA	0.409543	0.409543	2.78E-05	22.37284
177	184	rs7092291	T	C	T	C	-0.00986	-0.0585	0.412525	0.41	FALSE	FALSE	FALSE	9MV276	0.1594	0.7136	28243	outcome	TRUE	reported	textfile	15	1982	10	9998041	0.002	7.74E-10	805431	exposure	TRUE	reported	iBwP7P	textfile	2	TRUE	NA	0.587475	0.412525	3.02E-05	24.30484
178	186	rs71367545	A	G	A	G	0.0124	0.1828	0.161034	0.16	FALSE	FALSE	FALSE	9MV276	0.1965	0.3522	28243	outcome	TRUE	reported	textfile	86	7713	18	79816337	0.002	1.70E-10	805431	exposure	TRUE	reported	iBwP7P	textfile	2	TRUE	NA	0.838966	0.161034	4.77E-05	38.4399
179	187	rs71491832	G	C	G	C	-0.0183	-0.2845	0.074553	0.07	FALSE	TRUE	FALSE	9MV276	0.2924	0.3305	28243	outcome	TRUE	reported	textfile	18	2838	11	1.25E+08	0.003	3.72E-10	805431	exposure	TRUE	reported	iBwP7P	textfile	2	TRUE	NA	0.925447	0.074553	4.62E-05	37.20991
180	188	rs71627577	G	A	G	A	-0.017	-0.0407	0.119284	0.12	FALSE	FALSE	FALSE	9MV276	0.2482	0.8698	28243	outcome	TRUE	reported	textfile	192	20700	5	43125693	0.002	5.15E-12	805431	exposure	TRUE	reported	iBwP7P	textfile	2	TRUE	NA	0.880716	0.119284	8.97E-05	72.24982
181	189	rs7195043	T	C	T	C	-0.00883	0.0377	0.444334	0.45	FALSE	FALSE	FALSE	9MV276	0.1582	0.8117	28243	outcome	TRUE	reported	textfile	70	6533	16	89954453	0.002	2.13E-08	805431	exposure	TRUE	reported	iBwP7P	textfile	2	TRUE	NA	0.555666	0.444334	2.42E-05	19.49218
182	190	rs7205551	A	G	A	G	0.0102	0.0326	0.434394	0.43	FALSE	FALSE	FALSE	9MV276	0.1563	0.8349	28243	outcome	TRUE	reported	textfile	66	6090	16	69572065	0.002	1.15E-09	732296	exposure	TRUE	reported	iBwP7P	textfile	2	TRUE	NA	0.565606	0.434394	3.55E-05	26.00993
183	191	rs7224742	T	C	T	C	-0.011	-0.2286	0.633201	0.63	FALSE	FALSE	FALSE	9MV276	0.1607	0.1549	28243	outcome	TRUE	reported	textfile	72	6682	17	32330039	0.002	1.02E-11	805431	exposure	TRUE	reported	iBwP7P	textfile	2	TRUE	NA	0.366799	0.366799	3.76E-05	30.24992
184	192	rs72664906	C	T	C	T	0.0143	-0.0183	0.119284	0.12	FALSE	FALSE	FALSE	9MV276	0.2226	0.9344	28243	outcome	TRUE	reported	textfile	104	8163	1	31704540	0.002	1.85E-09	748334	exposure	TRUE	reported	iBwP7P	textfile	2	TRUE	NA	0.880716	0.119284	6.83E-05	51.12236
185	193	rs72733235	C	T	C	T	0.0127	0.107	0.177932	0.18	FALSE	FALSE	FALSE	9MV276	0.2213	0.6287	28243	outcome	TRUE	reported	textfile	246	27851	9	38275775	0.002	1.86E-09	805431	exposure	TRUE	reported	iBwP7P	textfile	2	TRUE	NA	0.822068	0.177932	5.01E-05	40.3224
186	194	rs72789627	T	C	T	C	-0.0171	-0.0072	0.134195	0.13	FALSE	FALSE	FALSE	9MV276	0.234	0.9756	28243	outcome	TRUE	reported	textfile	183	19738	5	1.07E+08	0.002	1.54E-13	805431	exposure	TRUE	reported	iBwP7P	textfile	2	TRUE	NA	0.865805	0.134195	9.08E-05	73.10232
187	195	rs72904370	C	G	C	G	-0.0108	-0.2605	0.217694	0.22	FALSE	TRUE	FALSE	9MV276	0.1895	0.1693	28243	outcome	TRUE	reported	textfile	23	3325	11	46018464	0.002	1.59E-08	805431	exposure	TRUE	reported	iBwP7P	textfile	2	TRUE	NA	0.782306	0.217694	3.62E-05	29.15993

188	196	rs7333559	A	G	A	G	-0.014	0.0937	0.791252	0.79	FALSE	FALSE	FALSE	9MV276	0.1887	0.6195	28243	outcome	TRUE	reported	textfile	43	4563	13	99894196	0.002	3.24E-13	805431	exposure	TRUE	reported	iBwP7P	textfile	2	TRUE	NA	0.208748	0.208748	6.08E-05	48.99988
189	197	rs745570	G	A	G	A	-0.0104	0.0024	0.49503	0.49	FALSE	FALSE	FALSE	9MV276	0.1559	0.988	28243	outcome	TRUE	reported	textfile	77	7264	17	79807926	0.002	8.70E-11	780334	exposure	TRUE	reported	iBwP7P	textfile	2	TRUE	NA	0.50497	0.49503	3.47E-05	27.03993
190	199	rs76132272	T	C	T	C	0.018	0.1966	0.055666	0.06	FALSE	FALSE	FALSE	9MV276	0.2993	0.5114	28243	outcome	TRUE	reported	textfile	100	8008	1	2.27E+08	0.003	9.43E-09	748334	exposure	TRUE	reported	iBwP7P	textfile	2	TRUE	NA	0.944334	0.055666	4.81E-05	35.9999
191	200	rs7629352	G	A	G	A	0.00986	-0.1243	0.296223	0.3	FALSE	FALSE	FALSE	9MV276	0.1681	0.4598	28243	outcome	TRUE	reported	textfile	158	15764	3	16807327	0.002	7.82E-09	805431	exposure	TRUE	reported	iBwP7P	textfile	2	TRUE	NA	0.703777	0.296223	3.02E-05	24.30484
192	201	rs763053	C	T	C	T	-0.0149	0.1047	0.267396	0.26	FALSE	FALSE	FALSE	9MV276	0.1886	0.5787	28243	outcome	TRUE	reported	textfile	65	6057	16	685921	0.002	6.71E-16	799280	exposure	TRUE	reported	iBwP7P	textfile	2	TRUE	NA	0.732604	0.267396	6.94E-05	55.50236
193	202	rs76608582	A	C	A	C	-0.0267	0.5149	0.045726	0.04	FALSE	FALSE	FALSE	9MV276	0.3791	0.1744	28243	outcome	TRUE	reported	textfile	90	7774	19	4474728	0.004	2.76E-13	799016	exposure	TRUE	reported	iBwP7P	textfile	2	TRUE	NA	0.954274	0.045726	5.58E-05	44.55551
194	203	rs7666804	C	T	C	T	-0.0109	-0.0474	0.393638	0.39	FALSE	FALSE	FALSE	9MV276	0.1585	0.7649	28243	outcome	TRUE	reported	textfile	180	19364	4	56883982	0.002	1.18E-08	573529	exposure	TRUE	reported	iBwP7P	textfile	2	TRUE	NA	0.606362	0.393638	5.18E-05	29.7024
195	204	rs77307359	C	T	C	T	-0.0112	-0.146	0.21173	0.21	FALSE	FALSE	FALSE	9MV276	0.197	0.4586	28243	outcome	TRUE	reported	textfile	179	19190	4	34838145	0.002	2.07E-08	805431	exposure	TRUE	reported	iBwP7P	textfile	2	TRUE	NA	0.78827	0.21173	3.89E-05	31.35992
196	205	rs7788527	C	T	C	T	0.00952	-0.2171	0.686879	0.68	FALSE	FALSE	FALSE	9MV276	0.1654	0.1893	28243	outcome	TRUE	reported	textfile	226	25890	7	71114501	0.002	1.58E-08	805431	exposure	TRUE	reported	iBwP7P	textfile	2	TRUE	NA	0.313121	0.313121	2.81E-05	22.65754
197	206	rs7804551	G	A	G	A	-0.0152	0.1069	0.163022	0.16	FALSE	FALSE	FALSE	9MV276	0.2162	0.621	28243	outcome	TRUE	reported	textfile	230	26024	7	99521487	0.002	1.09E-12	805431	exposure	TRUE	reported	iBwP7P	textfile	2	TRUE	NA	0.836978	0.163022	7.17E-05	57.75986
198	207	rs78175438	C	T	C	T	0.0162	0.2166	0.11829	0.12	FALSE	FALSE	FALSE	9MV276	0.2424	0.3716	28243	outcome	TRUE	reported	textfile	122	11331	21	39291329	0.002	2.02E-11	805431	exposure	TRUE	reported	iBwP7P	textfile	2	TRUE	NA	0.88171	0.11829	8.15E-05	65.60984
199	208	rs7829715	C	T	C	T	-0.0117	0.1677	0.560636	0.56	FALSE	FALSE	FALSE	9MV276	0.1565	0.2839	28243	outcome	TRUE	reported	textfile	234	26226	8	58891277	0.002	1.30E-13	805431	exposure	TRUE	reported	iBwP7P	textfile	2	TRUE	NA	0.439364	0.439364	4.25E-05	34.22242
200	209	rs7830359	T	C	T	C	-0.0103	0.0309	0.350895	0.35	FALSE	FALSE	FALSE	9MV276	0.1955	0.8744	28243	outcome	TRUE	reported	textfile	231	26068	8	10976748	0.002	3.88E-09	697342	exposure	TRUE	reported	iBwP7P	textfile	2	TRUE	NA	0.649105	0.350895	3.80E-05	26.52242
201	210	rs79222572	G	T	G	T	0.0103	0.1471	0.255467	0.26	FALSE	FALSE	FALSE	9MV276	0.1802	0.4142	28243	outcome	TRUE	reported	textfile	205	22526	6	1.65E+08	0.002	1.53E-08	805431	exposure	TRUE	reported	iBwP7P	textfile	2	TRUE	NA	0.744533	0.255467	3.29E-05	26.52243
202	211	rs7929518	G	A	G	A	0.0116	0.4139	0.772366	0.77	FALSE	FALSE	FALSE	9MV276	0.1854	0.02558	28243	outcome	TRUE	reported	textfile	28	3401	11	86269916	0.002	9.06E-10	805431	exposure	TRUE	reported	iBwP7P	textfile	2	TRUE	NA	0.227634	0.227634	4.18E-05	33.63992
203	212	rs7947391	G	A	G	A	0.00929	0.1122	0.577535	0.57	FALSE	FALSE	FALSE	9MV276	0.1578	0.4771	28243	outcome	TRUE	reported	textfile	25	3375	11	66419411	0.002	7.55E-09	805431	exposure	TRUE	reported	iBwP7P	textfile	2	TRUE	NA	0.422465	0.422465	2.68E-05	21.57597
204	213	rs7969559	G	A	G	A	-0.011	0.2541	0.681909	0.68	FALSE	FALSE	FALSE	9MV276	0.1739	0.144	28243	outcome	TRUE	reported	textfile	34	3806	12	69261387	0.002	3.28E-10	805431	exposure	TRUE	reported	iBwP7P	textfile	2	TRUE	NA	0.318091	0.318091	3.76E-05	30.24992
205	214	rs7984311	A	G	A	G	0.00921	0.2197	0.387674	0.38	FALSE	FALSE	FALSE	9MV276	0.1624	0.1761	28243	outcome	TRUE	reported	textfile	35	3828	13	1E+08	0.002	1.77E-08	805431	exposure	TRUE	reported	iBwP7P	textfile	2	TRUE	NA	0.612326	0.387674	2.63E-05	21.20597
206	215	rs7986094	C	A	C	A	0.00988	0.1304	0.694831	0.69	FALSE	FALSE	FALSE	9MV276	0.1718	0.448	28243	outcome	TRUE	reported	textfile	36	3838	13	30455794	0.002	5.99E-09	805431	exposure	TRUE	reported	iBwP7P	textfile	2	TRUE	NA	0.305169	0.305169	3.03E-05	24.40354
207	216	rs8001839	G	A	G	A	0.0094	0.0837	0.323062	0.32	FALSE	FALSE	FALSE	9MV276	0.1639	0.6096	28243	outcome	TRUE	reported	textfile	42	4560	13	96457819	0.002	3.74E-08	780334	exposure	TRUE	reported	iBwP7P	textfile	2	TRUE	NA	0.676938	0.323062	2.83E-05	22.08994
208	217	rs8067305	A	G	A	G	0.011	-0.0972	0.574553	0.57	FALSE	FALSE	FALSE	9MV276	0.1601	0.5437	28243	outcome	TRUE	reported	textfile	71	6545	17	2072363	0.002	4.95E-12	805431	exposure	TRUE	reported	iBwP7P	textfile	2	TRUE	NA	0.425447	0.425447	3.76E-05	30.24992
209	218	rs8069451	C	T	C	T	0.0112	0.0641	0.246521	0.25	FALSE	FALSE	FALSE	9MV276	0.181	0.7231	28243	outcome	TRUE	reported	textfile	73	6817	17	39348680	0.002	1.02E-09	805431	exposure	TRUE	reported	iBwP7P	textfile	2	TRUE	NA	0.753479	0.246521	3.89E-05	31.35992
210	219	rs846781	C	T	C	T	-0.0115	-0.0147	0.737575	0.73	FALSE	FALSE	FALSE	9MV276	0.1726	0.9321	28243	outcome	TRUE	reported	textfile	198	21979	6	1.01E+08	0.002	4.80E-11	805431	exposure	TRUE	reported	iBwP7P	textfile	2	TRUE	NA	0.262425	0.262425	4.10E-05	33.06242
211	220	rs853684	C	T	C	T	-0.0116	-0.0488	0.380716	0.4	FALSE	FALSE	FALSE	9MV276	0.1753	0.7807	28243	outcome	TRUE	reported	textfile	206	23073	6	28326773	0.002	8.55E-13	805431	exposure	TRUE	reported	iBwP7P	textfile	2	TRUE	NA	0.619284	0.380716	4.18E-05	33.63992
212	221	rs888292	T	A	T	A	-0.0107	-0.2589	0.295229	0.29	FALSE	TRUE	FALSE	9MV276	0.1693	0.1263	28243	outcome	TRUE	reported	textfile	79	7286	18	27631008	0.002	2.03E-09	748334	exposure	TRUE	reported	iBwP7P	textfile	2	TRUE	NA	0.704771	0.295229	3.82E-05	28.62242
213	222	rs911781	G	A	G	A	-0.00878	0.0465	0.568588	0.56	FALSE	FALSE	FALSE	9MV276	0.1583	0.7691	28243	outcome	TRUE	reported	textfile	6	1293	10	1.22E+08	0.002	4.99E-08	780334	exposure	TRUE	reported	iBwP7P	textfile	2	TRUE	NA	0.431412	0.431412	2.47E-05	19.27205
214	223	rs9323328	G	A	G	A	-0.0108	0.1523	0.526839	0.53	FALSE	FALSE	FALSE	9MV276	0.1563	0.33	28243	outcome	TRUE	reported	textfile	47	4649	14	58186796	0.002	7.25E-12	805431	exposure	TRUE	reported	iBwP7P	textfile	2	TRUE	NA	0.473161	0.473161	3.62E-05	29.15993
215	224	rs9375371	A	G	A	G	0.013	0.0306	0.293241	0.29	FALSE	FALSE	FALSE	9MV276	0.173	0.8596	28243	outcome	TRUE	reported	textfile	213	23858	6	98303804	0.002	1.41E-13	805431	exposure	TRUE	reported	iBwP7P	textfile	2	TRUE	NA	0.706759	0.293241	5.25E-05	42.2499
216	225	rs9402093	T	G	T	G	0.0101	0.3462	0.691849	0.71	FALSE	FALSE	FALSE	9MV276	0.1693	0.0409	28243	outcome	TRUE	reported	textfile	201	22462	6	1.29E+08	0.002	3.38E-09	805431	exposure	TRUE	reported	iBwP7P	textfile	2	TRUE	NA	0.308151	0.308151	3.17E-05	25.50244
217	226	rs9423279	G	C	G	C	-0.0108	-0.1743	0.638171	0.63	FALSE	TRUE	FALSE	9MV276	0.166	0.2935	28243	outcome	TRUE	reported	textfile	7	1294	10	1.24E+08	0.002	9.64E-11	775111	exposure	TRUE	reported	iBwP7P	textfile	2	TRUE	NA	0.361829	0.361829	3.76E-05	29.15992
218	227	rs951740	A	G	A	G	0.0172	-0.1073	0.636183	0.64	FALSE	FALSE	FALSE	9MV276	0.1593	0.5006	28243	outcome	TRUE	reported	textfile	106	8343	1	43546066	0.002	3.78E-26	805431	exposure	TRUE	reported	iBwP7P	textfile	2	TRUE	NA	0.363817	0.363817	9.18E-05	73.95982
219	229	rs9538536	G	T	G	T	-0.00936	-0.029	0.668986	0.67	FALSE	FALSE	FALSE	9MV276	0.1674	0.8626	28243	outcome	TRUE	reported	textfile	38	4382	13	59962187	0.002	4.28E-08	805431	exposure	TRUE	reported	iBwP7P	textfile	2	TRUE	NA	0.331014	0.331014	2.72E-05	21.90235
220	230	rs9613472	G	A	G	A	0.00867	0.1095	0.541749	0.54	FALSE	FALSE	FALSE	9MV276	0.1617	0.4984	28243	outcome	TRUE	reported	textfile	124	11350	22	27576518	0.002	4.02E-08	805431	exposure	TRUE	reported	iBwP7P	textfile	2	TRUE	NA	0.458251	0.458251	2.33E-05	18.79218
221	231	rs9627272	C	G	C	G	-0.00991	0.0928	0.389662	0.38	FALSE	TRUE	FALSE	9MV276	0.1642	0.5721	28243	outcome	TRUE	reported	textfile	127	11605	22	46046408	0.002	1.28E-09	780334	exposure	TRUE	reported	iBwP7P	textfile	2	TRUE	NA	0.610338	0.389662	3.15E-05	24.55196
222	232	rs963354	A	C	A	C	0.0107	-0.016	0.704771	0.7	FALSE	FALSE	FALSE	9MV276	0.1667	0.9236	28243	outcome	TRUE	reported	textfile	157	15510	3	1.58E+08	0.002	2.35E-10	805431	exposure	TRUE	reported	iBwP7P	textfile	2	TRUE	NA	0.295229	0.295229	3.55E-05	28.62243
223	233	rs9679319	G	T	G	T	0.0175	0.0379	0.56163	0.56	FALSE	FALSE	FALSE	9MV276	0.1561	0.8079	28243	outcome	TRUE	reported	textfile	128	11835	2	1.04E+08	0.002	4.52E-23	640245	exposure	TRUE	reported	iBwP7P	textfile	2	TRUE	NA	0.43837	0.43837	0.00012	76.56226
224	234	rs9763225	G	A	G	A	0.0139	0.0929	0.26839	0.27	FALSE	FALSE	FALSE	9MV276	0.1723	0.5899	28243	outcome	TRUE	reported	textfile	194	20984	5	60961623	0.002	5.38E-12	624207	exposure	TRUE	reported	iBwP7P	textfile	2	TRUE	NA	0.73161	0.26839	7.74E-05	48.30235
225	235	rs993700	C	T	C	T	-0.0141	0.0339	0.767396	0.76	FALSE	FALSE	FALSE	9MV276	0.1864	0.8555	28243	outcome	TRUE	reported	textfile	181	19398	4	66960176	0.002	1.00E-13	805431	exposure	TRUE	reported	iBwP7P	textfile	2	TRUE	NA	0.232604	0.232604	6.17E-05	49.70238
226	236	rs9987376	G	T	G	T	-0.0137	0.1682	0.569583	0.57	FALSE	FALSE	FALSE	9MV276	0.159	0.2903	28243	outcome	TRUE	reported	textfile	238	26974	8	92177786	0.002	1.02E-17	805431	exposure	TRUE	reported	iBwP7P	textfile	2	TRUE	NA	0.430417	0.430417	5.83E-05	46.92238

Supplementary Table 2. Per-study descriptive statistics for trans-ancestry and ancestry-stratified meta-analyses.

		Smoking Initiation				Age of Initiation of Smoking					Cigarettes per Day[b,e]					Smoking Cessation			
		Phenotype Descriptives		Genomic Control Values		Phenotype Descriptives			Genomic Control Values		Phenotype Descriptives			Genomic Control Values		Phenotype Descriptives		Genomic Control Values	
**Ancestry**	**Study Name**	**N**	**% Ever Regular Smoker**	**MAF > 0.01**	**0.01<= MAF >0.001**	**N**	**Mean**	**Var**	**MAF > 0.01**	**0.01<= MAF >0.001**	**N**	**Mean**	**Var**	**MAF > 0.01**	**0.01<= MAF >0.001**	**N**	**% Current Smoker**	**MAF > 0.01**	**0.01<= MAF >0.001**
EUR	23andMe - EUR Males	866,242	43%	2.13	1.14	129,200	17.35	18.42	1.11	1.01	129,471	2.38	2.76	1.18	1.02	362,467	23%	1.18	1.04
EUR	23andMe - EUR Females	997,356	41%	1.93	1.13	165,955	16.95	18.80	1.11	1.02	162,521	2.30	2.30	1.22	1.03	396,492	25%	1.30	1.07
EUR	ALSPAC	11,345	42%	1.00	1.01	4,691	16.00	7.80	1.00	1.01	4,314	3.40	2.50	1.01	1.00	4,748	65%	1.00	0.99
EUR	ARIC - TOPMed	6,010	60%	1.04	1.09	3,485	18.72	23.80	1.00	0.90	3,494	2.97	1.17	1.01	0.92	3,575	41%	1.02	0.99
EUR	BEAGESS	4,293	65%	1.02	1.03											2,805	20%	1.02	1.07
EUR	BLS	1,238	46%	1.05	1.03	546	15.70	6.40	1.02	1.05	546	0.70	0.70	1.04	1.02	548	48%	1.03	1.00
EUR	CADD	1,192	85%	1.01	1.00	775	16.10	0.40	1.01	0.89	523	2.40	1.40	1.01	1.06	1,002	33%	1.01	0.95
EUR	COGEND					1,952	13.70	10.70	0.99	1.01	1,940	2.60	2.00	1.01	0.98	1,954	69%	1.02	1.01
EUR	COPDGene - TOPMed					6,520	17.02	17.12	0.96	0.87	6,520	3.43	0.91	0.99	0.80	6,520	39%	0.99	0.84
EUR	deCODE	57,097	70%	1.11	1.05	40,314	9.5[a]	8.4[a]	1.06	1.01	44,505	0.9[c]	0.7[c]	1.14	1.08	34,820	37%	1.08	1.06
EUR	EGCUT	48,038	42%	1.13	1.07	19,511	18.69	1.06	1.02	1.01	19,732	2.08	0.67	1.06	1.03	20,210	67%	1.04	1.03
EUR	eMERGE - Cataracts	3,873	49%	1.01	1.01											1,884	17%	1.01	1.01
EUR	eMERGE - PAD	1,350	74%	1.01	1.13											1,002	52%	1.03	1.18
EUR	FinnTwin 1	1,006	54%	1.01	1.03	509	16.10	4.60	1.00	1.03	498	2.20	0.60	1.02	0.99	498	76%	1.01	1.05
EUR	FinnTwin 2	8,259	48%	1.02	1.07	3,913	19.25	23.64	1.01	1.04	3,913	14.85	77.90	1.01	1.03	4,163	55%	1.01	1.02
EUR	Framingham - TOPMed	4,076	55%	1.01	1.00	1,399	17.63	9.99	1.00	0.99	1,588	2.80	1.33	0.99	1.00	2,241	59%	1.00	0.98
EUR	Genes for Good	6,151	39%	1.01	1.03	2,198	17.98	16.19	0.99	1.02	2,209	2.16	0.94	0.99	0.97	2,413	31%	1.02	1.09
EUR	GERA																		
EUR	Harvard - Affy	6,901	56%	1.01	1.02	2,160	19.70	15.21	1.00	1.00	3,788	2.82	1.31	1.01	1.00	3,883	22%	1.01	1.06
EUR	Harvard - Illumina	6,456	50%	1.01	1.03	2,390	19.11	11.69	1.01	0.99	3,168	2.69	1.24	1.02	1.01	3,222	22%	1.01	1.06
EUR	Harvard - HumanCore	7,829	44%	1.03	1.03	2,464	17.90	14.41	1.01	1.00	3,395	2.69	1.28	1.00	1.00	3,467	22%	1.01	1.05
EUR	Harvard - OmniExpress	7,819	54%	1.01	1.02	2,734	19.70	13.69	1.00	1.00	4,153	2.73	1.25	1.02	1.01	4,258	22%	1.02	1.05
EUR	Harvard - OncoArray	9,087	46%	1.03	1.03	3,296	18.32	12.65	1.00	1.01	4,051	2.62	1.22	1.01	1.01	4,137	24%	1.01	1.05
EUR	HRS	9,989	57%	1.03	1.02	5,585	17.70	32.70	1.00	0.99	5,306	2.90	1.80	1.00	1.00				
EUR	HUNT	66,716	57%	1.20	1.10	35,311	18.80	26.20	1.05	1.05	33,705	2.10	0.50	1.05	1.05	37,964	58%	1.15	1.10
EUR	MCTFR	6,181	45%	1.02	1.05						2,535	2.60	2.30	1.00	1.03	2,808	67%	1.00	1.02
EUR	MESA - TOPMed	1,963	56%	0.98	0.99						1,034	2.75	1.52	1.04	0.98				
EUR	METSIM	9,607	57%	1.01	1.02	1,500	22.90	70.70	0.99	1.01	1,507	2.30	0.70	1.01	1.00	5,504	72%	1.01	1.02
EUR	NESCOG	477	45%	1.04	1.57						210	2.10	0.80	1.02	1.02	216	27%	1.10	1.24
EUR	NAG-FIN	2,052	85%	0.99	1.00	1,704	17.70	20.50	1.00	1.01	1,716	2.80	0.90	0.99	1.00	1,649	49%	1.01	1.03
EUR	NTR	7,266	47%	1.01	1.06	2,955	18.00	18.10	1.01	1.02	2,725	2.40	0.80	1.00	1.01	3,107	38%	1.01	1.06
EUR	PAGE - CARDIA										685	15.18	10.65	1.02	NA				
EUR	QIMR					4,193	16.80	11.50	1.04	1.04	4,409	3.70	1.20	1.05	1.05				
EUR	SardiNIA	5,459	39%	1.00	1.03	2,057	21.00	40.50	1.00	1.01	2,105	2.50	1.20	1.01	1.03	2,105	54%	1.01	1.01
EUR	UKB	448,196	45%	1.56	1.07	147,551	17.19	11.46	1.20	1.08	143,301	2.70	0.87	1.24	1.10	199,612	17%	1.21	1.06
EUR	NINDS SiGN	3,665	43%	1.00	1.03											1,562	37%	1.00	1.10
EUR	FINRISK	25,097	51%	1.12	1.06	12,912	18.15	23.85	1.03	1.01	6,762	2.30	0.80	1.04	1.02	12,361	55%	1.05	1.03
EUR	WLS	8,496	58%	1.00	1.03	4,709	18.01	18.53	1.01	1.00	4,821	3.24	1.77	1.00	1.01	4,915	27%	1.00	1.01
EUR	Spit for Science	2,734	24%	1.03	1.01						585	1.43	0.51	0.99	0.98				
EUR	AMISH - TOPMed	846	30%	1.01	1.15	222	18.17	11.12	0.90	1.07	123	1.66	0.82	1.02	1.19	209	50%	0.94	1.15
EUR	CFS - TOPMed					228	17.56	23.38	1.05	1.09	228	2.67	1.24	1.08	0.95	228	41%	0.92	0.98
EUR	ECLIPSE - TOPMed					1,353	17.04	16.32	1.00	1.02	1,202	3.15	0.99	1.00	1.04	1,349	39%	1.01	1.01
EUR	GeneSTAR	913	44%	1.06	0.95	382	17.39	13.02	0.94	0.97	160	2.56	0.86	1.07	1.11	403	40%	0.95	1.05
EUR	GOLDN - TOPMed	849	28%	1.03	1.00	197	16.89	17.58	0.95	0.97	117	2.79	2.34	1.01	1.00	234	27%	0.97	0.97
EUR	Boston - TOPMed										64	3.61	1.04	1.03	0.96	64	17%	1.05	1.11

EUR	IPF - TOPMed	425	73%	0.98	0.96	125	18.42	30.29	1.02	1.00	126	2.88	1.18	1.01	1.02	275	11%	1.03	1.05
EUR	CHS - TOPMed	2,295	55%	1.03	0.84						1,219	2.70	1.19	0.91	0.81	1,264	20%	1.04	1.09
EUR	HCHS SOL - TOPMed										91	2.01	1.43	0.98	1.05	107	62%	1.00	1.04
EUR	HVH - TOPMed	626	47%	1.01	0.99											296	17%	1.00	1.02
EUR	JHS - TOPMed	48	29%	1.00	1.10														
EUR	VTE - TOPMed	348	64%	1.00	0.96											223	61%	1.00	1.00
EUR	WGHS - TOPMed	113	50%	1.03	1.00											57	23%	1.04	1.00
EUR	WHI - TOPMed	9,050	50%	1.08	1.49	3,545	19.70	15.56	1.02	1.13	3,424	2.66	1.37	1.00	1.05	4,451	15%	1.04	1.27
EUR	WHI - MOPMAP																		
EUR	WHI - HIPFX																		
EUR	WHI - GARNET																		
EUR	WHI - LLS																		
EUR	WHI - GECCO																		
EUR	WHI - WHIMS+																		
EUR TOTAL SAMPLE SIZE		2,669,029				618,541					618,489					1,147,272			

Table S3. Characteristics of source GWAS cohorts for exposure and outcome variables

Variable	Source	Sample Size	Available Descriptives
Smoking amount (cig/day)	GSCAN	618,489	• *None beyond summary statistics*
Smoking initiation	GSCAN	2,669,029	• Ever-smoker proportion: 53.7%
Age at smoking initiation	GSCAN	618,514	• Mean age: 17.2 years (SD=4.3)
Smoking cessation	GSCAN	1,147,272	• Former-smoker proportion: 38.2%
Laryngeal cancer	NCI GWAS	89 cases	• *None beyond case-control counts*
		28,154 controls	• Control age range: 55–74 years

Table S4. Power analysis results for Mendelian randomization estimates, demonstrating reduced sensitivity to detect moderate effect sizes (odds ratio <15) due to the limited number of laryngeal cancer cases (n=89). The analysis highlights the constraints in statistical power and the increased uncertainty around effect size precision.

Two-stage least squares

Power	0.06	
NCP	0.10	Non-Centrality-Parameter
F-statistic	1.72	The strength of the instrument

Power or sample size calculations for two-stage least squares Mendelian Randomization studies using a genetic instrument Z (a SNP or allele score), a continuous exposure variable X (e.g. body mass index [BMI, kgm2]) and a continuous outcome variable Y (e.g. blood pressure [mmHg]).

YZ association

Power	0.49	
NCP	3.79	Non-Centrality-Parameter

Power or sample size calculations for the regression association of a genetic instrument Z (e.g. a BMI SNP), with a continuous outcome variable Y (blood pressure).

Supplementary File 1. Complete R code for all Mendelian randomization analyses, provided for transparency and reproducibility.

```
"install.packages(""TwoSampleMR"")"

"setwd(""F:/…˙–≈∑÷Œˆ/≤®≤®"")#…Ë÷√¬∑æ∂"

library(TwoSampleMR)

"a<-read.table(""250.11_PheCode.v1.0.fastGWA.txt"",header = T) #∂¡»°±©¬∂Œƒº˛¥¢¥ÊŒ™a."

"b<-subset(a,p_value<5e-8)#,⋯∏—° P÷µ£¨¥¢¥ÊŒ™b"

"write.csv(b,file = ""exposure.csv"")#∏ƒ–¥bŒƒº˛∏Ò Ω£¨Õ¨ ±Ω´b∑≈ ‘⁄TwoSampleMRŒƒº˛º–¿Ô"

"c<-system.file(""exposure.csv"",package = ""TwoSampleMR"")#œµÕ≥¥¶¿Ì“ ‘⋯œµƒbŒƒº˛¥¢¥ÊŒ™C"

"D<-read_exposure_data(filename =c,sep="","",snp_col = ""variant_id"",beta_col = ""beta"",se_col =""standard_error"" ,effect_allele_col = ""effect_allele"",other_allele_col = ""other_allele"",eaf_col = ""eaf"",pval_col = ""p_value"")#∏≥÷µ"

"D_clumpd<-clump_data(D,clump_kb = 10000,clump_r2 = 0.001,clump_p1 = 1,clump_p2 = 1,pop = ""EUR"")#…æ≥˝¡¨À¯ΔΩ∫,µƒSNP"


"setwd(""F:/…˙–≈∑÷Œˆ/MR—ßœ∞/hnsc"")#…Ë÷√Ω•æ÷µƒ¬∑æ∂"

"a2<-read.table(""hnsc.txt"",header = T)#∂¡»°Ω·æ÷Œƒº˛√‚√˚a2"

"e<-merge(D_clumpd,a2,by.x=""SNP"",by.y=""variant_id"")#Ω´±©¬∂(D_clumpd)∫ÕΩ•æ÷Œƒº˛(a2)»°ΩªØ±£¥ÊŒ™e,by.xµ»”⁄±©¬∂µƒSNP√˚,by.y=Ω·æ÷µƒsnp√˚"
```

```
"write.csv(e,file = ""outcome.csv"")#Ω´Ω·æ÷Œƒº¸¥¢¥Êoutcome.csv"

"out_data<-read_outcome_data(snps =D_clumpd$SNP,filename = ""outcome.csv"",sep = "","",snp_col =""SNP"",beta_col = ""b"",se_col =
""se"",effect_allele_col = ""effect_allele"",other_allele_col = ""other_allele"",eaf_col = ""efa"",pval_col = ""p"")#Ω´Ω·æ÷Œƒº¸∏≥÷µ¥Ê℄™out°™data"

"dat<-harmonise_data(exposure_dat =D_clumpd,outcome_dat = out_data )#Ω´±©¬∂Œƒº¸”ÎΩ·æ÷Œƒº¸–≠Õ¨¥Ê℄™dat"

"write.csv(dat,file = ""harmonising.csv"")#¥¢¥Ê“‘…œΩ·π˚"

mr(dat)#MR∑÷Œˆdat

generate_odds_ratios(mr_res = mr(dat))#∂¸∑÷¿‡±‰¡ø◊™ªØor

"mr(dat,method_list = c(""mr_ivw"",""mr_raps"",""mr_weighted_median"",""mr_egger_regression""))#mrÀƒ÷÷∑÷Œˆ∑Ω∑®£¨raps≈‹≤ª≥ˆæÕ…
æµÙ"

"mr_scatter_plot(mr_results=mr(dat,method_list = c(""mr_ivw"",""mr_weighted_median"",""mr_egger_regression"")),dat)#mrΩ·π˚◊˜Õº"

mr_heterogeneity(dat)#“Ï÷ –‘ºÏ≤,

mr_funnel_plot(singlesnp_results = mr_singlesnp(dat))#“Ï÷–‘ø…”ªØ

mr_pleiotropy_test(dat)#∂‡–ß–‘ºÏ≤,

mr_leaveoneout(dat)#√Ù∏–– ‘ºÏ≤,

mr_leaveoneout_plot(leaveoneout_results = mr_leaveoneout(dat))#√Ù∏–– ‘ºÏ≤,ø…”ªØ


"a<-extract_instruments(outcomes =""ukb-e-1269_EAS"")#Ã·»°‘⁄œﬂ±©¬∂″æ›Œ™a"
```

```
"b<-extract_outcome_data(snps = a$SNP,outcomes = ""ebi-a-GCST90018678"")#Ã·»°‘⁄œflΩ·æ÷ ˝æ›Œ™b"

"dat<-harmonise_data(exposure_dat =a,outcome_dat =b)#Ω´±©¬∂Œƒº¸”ÎΩ·æ÷Œƒº¸–≠Õ ¨ ¥ÊŒ™dat"

"write.csv(dat,file = ""harmonising.csv"")#¥¢¥Ê“‘…œΩ·π˚"

mr(dat)#MR∑÷Œˆ dat

generate_odds_ratios(mr_res = mr(dat))#∂¸∑÷¿‡±‰¡ø◊™ªØor
```

Supplementary Figure 1: Experimental flow chart for the examination of the effect of smoking amount on the risk of laryngeal cancer.

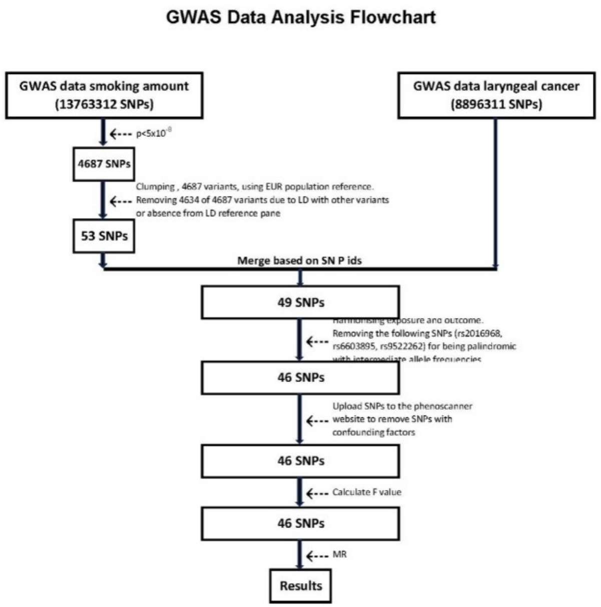

© 2025 Yang F. et al.
